# FGF family in health and disease

**DOI:** 10.1186/s43556-026-00429-0

**Published:** 2026-03-15

**Authors:** Xiaoyu Liu, Meiling Jing, Yueyi Yang, Qiaoqiao Jin, Bo Feng, Pengfei Zhang, Chenguang Niu, Xuchen Hu, Zhengwei Huang

**Affiliations:** 1 Department of Endodontics, Shanghai Ninth People’s Hospital, Shanghai Jiao Tong University School of Medicine, Shanghai, China; 2 College of Stomatology, Shanghai Jiao Tong University, Shanghai, China; 3 National Clinical Research Center for Oral Diseases, National Center for Stomatology; Shanghai Key Laboratory of Stomatology, Shanghai, 200001 China

**Keywords:** FGF, Canonical and non-canonical FGF signaling, Organ development and tissue homeostasis, Metabolic homeostasis, FGFR-targeted therapeutics

## Abstract

Fibroblast growth factors (FGFs) form an evolutionarily conserved signaling system that governs embryonic patterning, tissue regeneration, and systemic metabolic homeostasis. Through coordinated interactions with fibroblast growth factor receptors (FGFRs) and context-specific cofactors, FGF signaling enables precise spatial and temporal control of cellular fate and interorgan communication. While canonical FGFs coordinate local tissue dynamics, endocrine members like FGF19, FGF21, and FGF23 function as systemic hormones to regulate bile acid, glucose, and phosphate metabolism. Despite rapid advances in understanding these pathways, a unified framework that integrates their structural diversity, complex regulatory mechanisms, and the contrasting roles they play in health and disease remains fragmented. In this review, we systematically summarize the classification, structural features, and receptor specificity of the FGF family, with particular emphasis on canonical, endocrine, and intracellular FGFs. We delineate canonical and non-canonical FGF signaling pathways and their multilayered regulation by heparan sulfate proteoglycans, Klotho coreceptors, and intracellular feedback mechanisms. Furthermore, we integrate emerging insights into the roles of FGFs in organ development, tissue repair, metabolic regulation, and disease pathogenesis. A core translational insight emphasized throughout is the therapeutic duality of targeting the FGF axis: harnessing FGF agonism for tissue regeneration and metabolic regulation, versus employing FGF antagonism to block oncogenic signaling in cancer. By providing an integrated and mechanistic overview, this review clarifies key knowledge gaps and establishes a conceptual foundation for future FGF-based therapeutic innovation.

## Introduction

The fibroblast growth factor (FGF) family comprises 22 structurally similar proteins. Precise regulation of this family is vital for sustaining life, as it is deeply involved in processes ranging from embryonic tissue and organ formation to adult tissue repair, angiogenesis, and the maintenance of key metabolic homeostasis [1, 2]. FGFs can be classified into canonical, endocrine, and intracellular subtypes, with endocrine FGFs acting as long-distance messengers via the bloodstream to underscore the family’s role as a master regulator of interorgan signaling [3].

The study of the FGF axis remains a cutting-edge field, with recent breakthroughs in its complex signaling networks and clinical translation [4]. Engineered FGFs have been identified as a potentially effective therapeutic approach for metabolic disorders, including diabetes and obesity [5]. The advent of structural biology techniques has facilitated the redesign of FGFs [6]. In the field of cancer research, there is a growing focus on the development of targeted therapies, such as the use of fibroblast growth factor receptor (FGFR) inhibitors, which function by obstructing aberrant FGF signaling pathways [7, 8]. This approach has the potential to reveal novel therapeutic strategies for the management of tumors [9].

The review aims to guide readers from fundamental science to clinical therapies. First, the FGF structures, classifications, and their receptors are described. Next, an exploration of the regulatory functions of FGF in both healthy and diseased states is conducted. Finally, the novel therapeutic treatments targeting FGF pathway were discussed. By establishing connections between key concepts in the fields of biology and medicine, this review offers a comprehensive overview of FGF, encompassing molecular mechanisms and clinical therapies. The review provides a systematic foundation for deepening our understanding of the functions and potential of the FGF family.

## Structure and classification of the FGF family

### Subfamilies and members

**Fig. 1:**
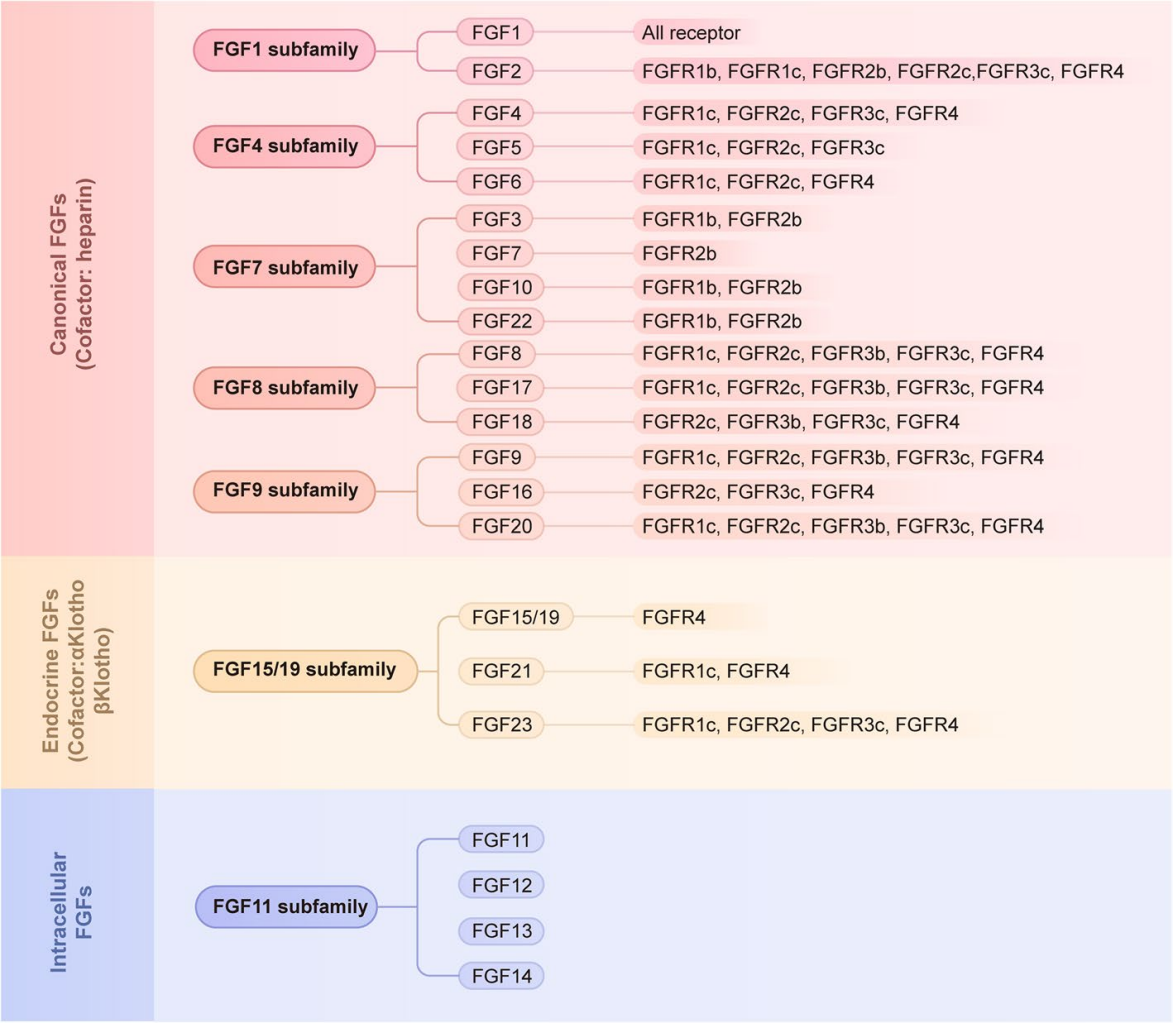
Classification of FGFs. Classification of fibroblast growth factors and their corresponding receptors. The FGF family comprises 22 members, grouped into three functional categories: canonical (secreted), endocrine, and intracellular FGFs. Further classification reveals seven subfamilies characterized by their biochemical functions, sequence homology, and evolutionary affinities

The FGF family is known to comprise 22 evolutionarily related members with a molecular weight of 17 to 34 kDa [10]. FGF1 to FGF23 are classified into three distinct groups according to their functional modes: canonical, endocrine, and intracellular FGFs. The human and mouse FGF families separately exclude FGF15 and FGF19, which are orthologs of one another. Additionally, based on the functional properties, phylogeny, and sequence similarities, the FGF family can be further categorized into 7 subfamilies: FGF1, FGF4, FGF7, FGF8, FGF9, FGF11, and FGF15/19 subfamilies. Each FGF subfamily consists of 2–4 FGF members. Most members of FGF family (including FGF1-10, FGF16-18, FGF20, and FGF22) are classified as secreted canonical FGFs. These members can be categorized into five paracrine subfamilies: the FGF1 subfamily (FGF1 and FGF2), the FGF4 subfamily (FGF4, FGF5, and FGF6), the FGF7 subfamily (FGF3, FGF7, FGF10, and FGF22), the FGF8 subfamily (FGF8, FGF17, FGF18), and the FGF9 subfamily (FGF9, FGF16, and FGF20) (Fig. 1) [11]. The canonical FGFs function as autocrine or paracrine agents, binding and activating the high-affinity tyrosine kinase receptors encoded by four genes of *FGFR1*, *FGFR2*, *FGFR3*, and *FGFR4*, as well as a truncated form of FGFR without an intracellular domain, known as *FGFRL1* (Fig. 1) [12, 13]. In addition to the canonical FGFs, three FGF members (FGF19, FGF21, and FGF23) are designated as the endocrine FGF subfamily, also known as the FGF15/19 subfamily (Fig. 1) [14]. With a weak affinity for the cognate FGF receptors, the endocrine FGFs enter the circulation and assert functions like hormones in distal organs, thereby playing crucial role in maintaining the systemic metabolic homeostasis [5, 15]. The remaining four members of the FGF family, FGF11 to FGF14, constitute the FGF11 subfamily (Fig. 1). These FGF members are non-signaling proteins that function in an intracrine manner, serving as coreceptors for voltage gated sodium channels or other molecules [12].

### Structural features

The FGFs range in size from 150 to 300 amino acids and shares a highly similar core structure motif [16]. The sequence analysis discovered a conserved sequence of 120 amino acids exhibiting sequence identities ranging from 16 to 65% [17]. The canonical FGFs are characterized by a globular β-trefoil fold composed of 12 antiparallel β-strands (β1–12), which is essential for their binding to FGF receptors and determining their biological activity. In contrast, the endocrine FGFs exhibit an atypical β-trefoil fold owing to the absence of β11 strand [18–20]. Moreover, the conversed core structure of FGFs is flanked by amino-terminal and carboxy-terminal regions highly diverse in length and sequence, conferring variable functions on different FGFs [20].

Paracrine and endocrine FGFs exert bioactive functions through binding and activating the FGFRs. During the canonical FGFs-induced signaling transduction, heparin or heparan sulfate proteoglycans (HSPGs) are the essential cofactors, which are glycoproteins with covalently attached heparan sulfate (HS) chains. HS is a glycosaminoglycan composed of repeating disaccharide units including glucuronic acid and amino acetylglucosamine [21]. The heterogeneous sulfation occurs at the 2-O position of glucuronic acid and the 6-O position of amino acetylglucosamine [22]. Notably, the paracrine FGFs have a heparin binding domain, thereby exhibiting moderate to high affinity for HS. The structural study unveiled that the HS-binding region was discretely localized within the β1-β2 loop and the truncated β10-β12 region, where the basic amino acids and backbone atoms are exposed for HS binding [23]. Although variation events occur on the primary sequence of HS binding site, the paracrine FGFs possess a shared topology due to the presence of conserved GXXXXGXXS/T motif, known as the glycine box [18, 19, 24]. However, endocrine FGFs exhibit significant differences in the topology of HS binding sites due to the absence of glycine box and the lack of the extended β10-β12 region. These structural differences result in low affinity of endocrine FGFs for HS, thereby allowing these FGF members enter the bloodstream and function as hormones in distant organs [16].

### FGF receptors

FGFRs are tyrosine kinases receptors comprising four members, FGFR1 to FGFR4, with gene sequences possessing high identities ranging from 56 to 71% [25]. Despite the high commonality, FGFRs differ a lot in terms of the ligand affinity. Structurally, FGFRs consist of three extracellular immunoglobulin domains (D1-D3), a single-pass transmembrane domain and a cytoplasmic tyrosine kinase domain. The extracellular ligand-binding domain comprises three immunoglobulin-like domains of D1, D2, and D3 [2, 19]. A distinctive serine-rich sequence with 7 to 8 acidic residues, also termed as the acid box, is located in the linker region connecting D1 and D2. During the function of FGFR, both acid box and D1 domain are responsible for receptor self-inhibition, while the D2 and D3 domains primarily take charge of the ligand-binding and specificity [26]. The transmembrane domain anchors the receptors in cell membrane and, together with the neighboring regions, facilitates receptor dimerization. The split cytoplasmic domain is involved in the transmitting of FGF/FGFR signals [20, 27]. Moreover, tissue-specific alternative splicing events and translational initiation in D3 domain of *FGFR1*, *FGFR2*, and *FGFR3* generate b and c variants, bearing distinct binding specificities for FGFs at different tissues. Therefore, there are seven FGFR isoforms in total, including FGFR1b, FGFR1c, FGFR2b, FGFR2c, FGFR3b, FGFR3c, and FGFR4 (Fig. 1) [28, 29].

Four members of the FGF11 subfamily are intracellular proteins that do not interact with extracellular receptors [11]. In contrast, the remaining eighteen FGFs, including both canonical and endocrine FGFs, function as ligands that bind to FGFRs [11]. The FGF/FGFR binding triggers the conformational changes of FGFRs, resulting in the dimerization and activation of downstream signaling cascade [20]. The initiation of FGF/FGFR signaling requires the specific assembly of ternary complexes, including the ligands, receptors, and cofactors [1]. Owing to the structural features, the canonical FGFs possess moderate to high affinity for the HS cofactors. However, differing from the canonical FGFs, the endocrine FGFs lack the glycine box and β10-β12 region, thereby exhibiting low binding affinity to HS chains [16]. The binding of endocrine FGFs to cognate FGFRs require cofactors of α-Klotho and β-Klotho [14]. Specifically, α-Klotho augments the FGF23 signaling by binding to FGFR1c, while β-Klotho facilitates the FGF19-induced signaling through binding to FGFR4, and FGF21-induced signaling through FGFR1c [18, 25, 30].

## FGF signaling pathways

### Canonical signaling pathways

**Fig. 2:**
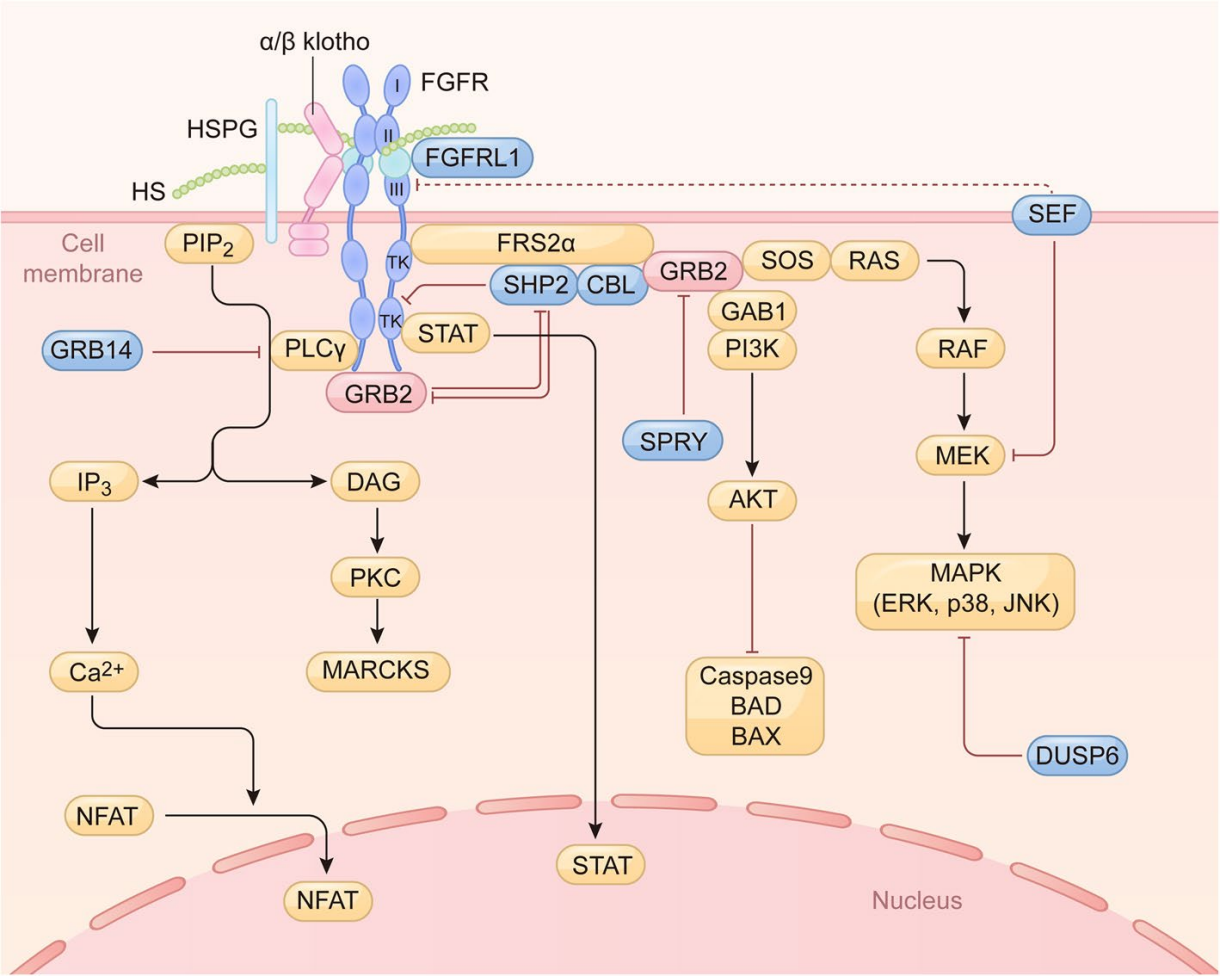
The canonical FGF signaling pathways. Binding of canonical FGFs to FGFR with HS (or HSPG) or α/β Klotho as a cofactor induces the formation of ternary FGF-FGFR-HS complex, which activates the FGFR intracellular tyrosine kinase domain by phosphorylation of specific tyrosine residues. The activated FGFR phosphorylated FRS2α, which then recruits GRB2. Activated GRB2 binds to SOS to activate RAS/RAF/MAPKs, including ERK MAPK, p38 MAPK, and JNK MAPK. The Activated GRB2 also binds GAB1 to activate PI3K/AKT, leading to inactivation of Caspase 9, BAD, and BAX. The activated FGFR can also recruit and activate PLCγ, which catalyzes the hydrolysis of PIP_2_ into IP_3_ and DAG. IP_3_ induces calcium ion release from intracellular stores and the nuclear translocation of NFAT. DAG activates the enzyme PKC and its downstream substrate MARCKS. FGFR kinase also activates STAT to regulate gene expression in the nucleus. FGFRL1 inhibits FGF signaling by acting as a decoy receptor of FGF. SHP2 inhibits the phosphorylation of FGFR and GRB2, while GRB2 can impede SHP2 phosphorylation. CBL acts as a negative regulator by promoting the degradation of FGFR and FRS2α. SPRY inhibits FGF signaling by interacting with GRB2. SEF inhibits MEK activation and FGFR phosphorylation. DUSP6 inhibits FGF signaling by dephosphorylating MAPK. GRB14 inhibits activation of PLCγ. FGF: fibroblast growth factor. FGFR: fibroblast growth factor receptor. HS: heparan sulfate. HSPG: heparin or heparan sulfate proteoglycans. FRS2α: FGFR substrate 2α. GRB2: growth factor receptor-bound 2. SOS: son of sevenless. MAPK: mitogen-activated protein kinases. GAB1: GRB2-associated binding protein 1. BAD: BCL-2 antagonist of cell death. BAX: BCL-2 associated X protein. PLCγ: phospholipase C-gamma. PIP_2_: phospholipid phosphatidylinositol-4, 5-bisphosphate. IP_3_: inositol-1,4,5-trisphosphate. DAG: diacylglycerol. PKC: protein kinase C. MARCKS: myristoylated Ala-rich C kinase substrate. STAT: signal transducer and activator of transcription. SHP2: Src homology region 2-containing protein tyrosine phosphatase 2. CBL: casitas B-lineage lymphoma proto-oncogene. SPRY: Sprouty. SEF: Similar expression to FGF. DUSP6: dual-specificity phosphatase 6. GRB14: Growth factor receptor bound protein 14

The canonical FGF signaling pathways are initiated upon the interaction of FGF ligands with the cognate FGFRs, including FGFR1 to FGFR4. The FGF-FGFR binding triggers the conformational alternation of receptors, leading to receptor dimerization and specific phosphorylation of seven tyrosine residues within the cytosolic tyrosine kinase domain [31, 32]. The intracellular phosphorylated residues, serving as the docking sites, in turn recruit and activate the downstream substrates by phosphorylation, triggering distinct signaling pathways and diverse cellular events (Fig. 2) [31, 32].

FGFR substrate 2α (FRS2α) and phospholipase C-gamma (PLCγ) are identified as the major docking proteins of the FGFR kinase [33]. FRS2α is a protein containing phosphotyrosine binding domain, mainly regulating the activation of mitogen-activated protein kinases (MAPK) and phosphatidylinositol-3 kinase/protein kinase B (Pl3K/AKT) signaling pathways [34–37]. The phosphorylated FRS2α recruits and produces the binding sites for the SH2-domain-containing adaptor protein, growth factor receptor-bound 2 (GRB2). Subsequently, GRB2 recruits either the guanine nucleotide exchange factor, son of sevenless (SOS), or the GRB2-associated binding protein 1 (GAB1) to form the signaling complex [34]. The SOS signaling complex then activates the downstream RAS GTPase and induces the transduction of RAS/RAF/MEK/MAPKs cascade including ERK1/2 MAPK, p38 MAPK, and JNK MAPK, which are associated with cell survival, cell proliferation, cell migration, cell differentiation, and other cellular events such as inflammatory responses [17, 38–40]. On the other hand, the GAB1 signaling complex induces the Pl3K mediated activation of AKT kinase, which then leads to the inactivation of pro-apoptotic factors, including caspase 9, BCL-2 antagonist of cell death (BAD), and BCL-2 associated X protein (BAX) (Fig. 2) [41–43].

In addition, the FGFR kinase-induced phosphorylation of SH2 domain-containing PLCγ initiates another distinct signaling pathway [44]. The activated PLCγ in turn catalyzes the hydrolysis of phospholipid phosphatidylinositol-4, 5-bisphosphate into inositol-1,4,5-trisphosphate (IP_3_) and diacylglycerol (DAG) [45]. IP_3_ induces the release of calcium ions from intracellular storage, which facilitates the activation of calcineurin and then promotes the nuclear translocation of nuclear factor of activated T cells (NFAT), which is responsible for expressing the productions associated with cell motility [46]. On the other hand, DAG activates the protein kinase C (PKC), which contributes to triggering the phosphorylation of downstream substrates known as myristoylated Ala-rich C kinase substrate (MARCKS), which contributes to cell motility [47]. In addition, FGFRs also activate the downstream of signal transducer and activator of transcription (STAT). In response to FGF1, STAT1 is activated in the primary growth plate chondrocytes, resulting in the inhibition of cell proliferation (Fig. 2) [48, 49].

### Non-canonical signaling

Beyond the canonical kinase-dependent pathways, FGFs can signal through mechanisms that are independent of FGFR kinase activity or, in some cases, even of FGF ligand binding [50]. Non-canonical FGF signaling, mediated through receptors such as syndecans, integrins, neural cell adhesion molecule (NCAM), and N-cadherin, regulates critical cellular processes including migration, adhesion, angiogenic activation, and signal specificity, thereby expanding the functional diversity and contextual responses of the FGF system beyond the classical tyrosine kinase receptor pathway [50]. The intracellular FGF subfamily (FGF11-FGF14) operates in a strictly kinase-independent manner, directly regulating the gating and trafficking of voltage-gated sodium channels within the cell [51].

**Fig. 3:**
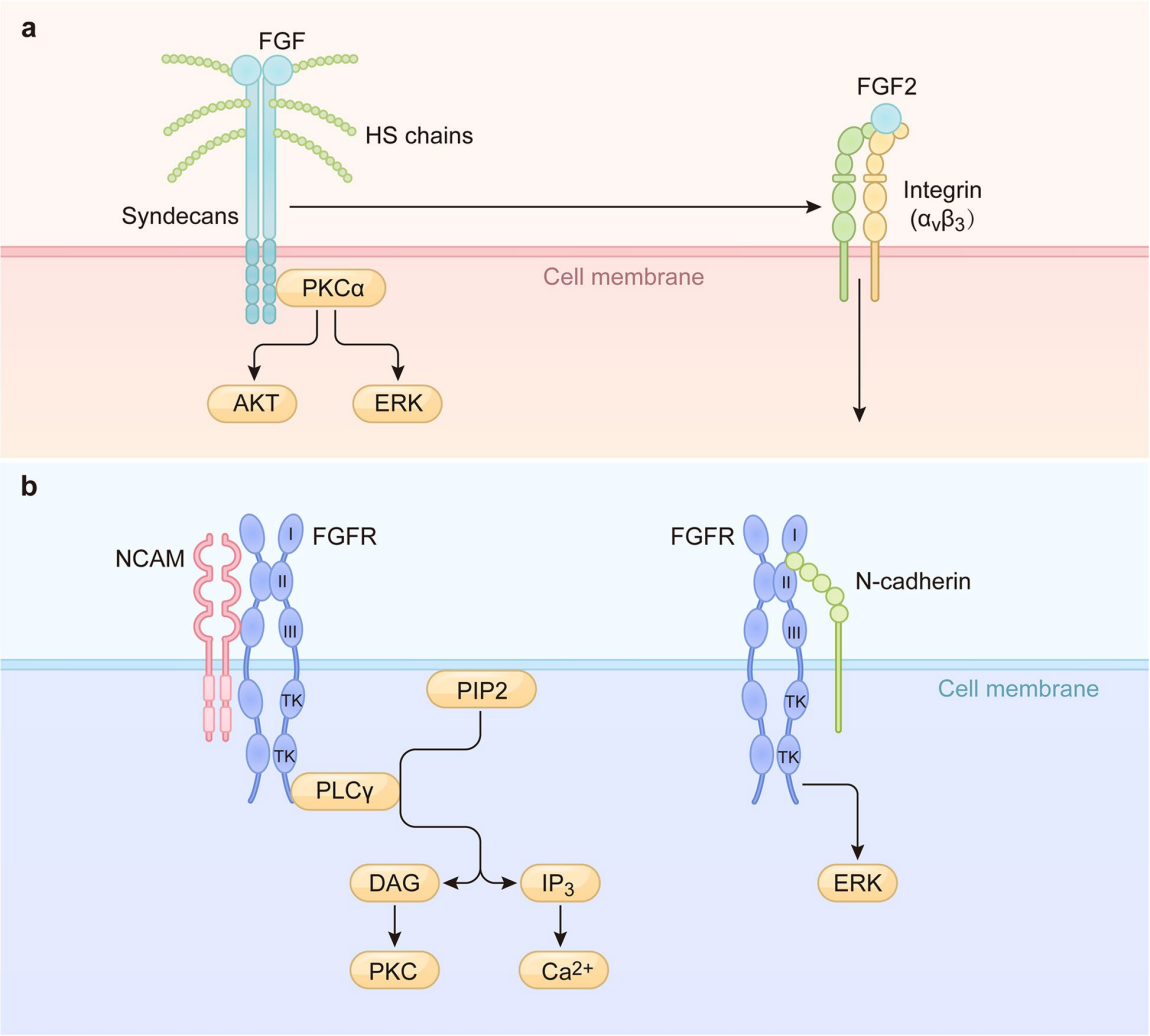
The non-canonical FGF signaling pathways. **a** Ligand (FGF)-dependent pathway. Syndecans can bind and trigger FGF-induced signaling via the HS chain, for example by activating PKCα to influence AKT and ERK. The αvβ3 integrin can also directly bind FGF2 to trigger downstream signaling pathways. Syndecans has been demonstrated to regulate integrin function. **b** Ligand-independent pathway. NCAM and N-cadherin initiate signaling through heterodimeric interactions with FGFR. NCAM-induced PLCγ activation depends on FGFR. The interaction between N-cadherin and FGFR is sufficient to trigger FGFR signaling, but it also causes FGFR retention at the cell surface, thereby enhancing its responsiveness to FGF. NCAM: Neural cell adhesion molecule

Non-canonical signaling can also be initiated in a ligand-dependent manner through receptors other than FGFRs. Syndecans, a family of HSPG, function as non-tyrosine kinase receptors of FGF ligands [52]. They bind FGF ligands through their HS chains. Despite the relatively low affinity of FGFs, syndecans have abundant expression on the plasma membrane and exhibit strong impact on the FGF ligands, inducing the downstream signaling [52]. For instance, FGF2 binding to syndecan-4 activates protein kinase Cα (PKCα), influencing processes like vascular cell mineralization [53]. Syndecans can also facilitate the nuclear translocation of FGF2 [54]. Integrins, such as αvβ3, can directly bind FGF2 on the cell surface, activating pathways that promote angiogenesis in endothelial cells [55–57]. The signaling specificity in these contexts is often determined by the composition of the receptor complex (e.g., crosstalk between syndecans and integrins) rather than a unique downstream cascade (Fig. 3) [50, 58].

In a ligand-independent manner, other cell surface molecules can directly engage and activate FGFRs. The NCAM contains a fibronectin type III (FNIII) peptide that binds FGFR1, stimulating pathways primarily associated with neuronal survival and neurite outgrowth. This interaction can activate PLCγ, increase intracellular calcium, and promote PKC phosphorylation [59–61]. Similarly, N-cadherin directly interacts with the extracellular domain of FGFR1. This interaction not only triggers downstream signaling implicated in processes like tumor metastasis but also sustains signaling by retaining FGFR1 at the plasma membrane and inhibiting its internalization, leading to prolonged ERK activation (Fig. 3) [62, 63].

### Regulation of FGF signaling

FGF signaling is intricately orchestrated by both positive and negative regulators at different levels to exert diverse biological functions. The initiation of effective signals and the specificity of these signals are highly dependent on cofactors and accessory proteins located on the cell surface. HSPGs, acting as key cofactors for paracrine FGF signaling, have been demonstrated to determine the initiation of the FGF signaling pathway [64]. As for the endocrine FGFs, which lack the structure of paracrine-conserved glycine box and β10-β12 domain, these FGF members exhibit low affinity for the HS chains, leading to disturbed FGF binding and FGFR activation. α/β Klotho serves as the mandatory cofactor, collaborating the c-splice isoforms of FGFR1 to FGFR3 as well as FGFR4 to facilitate the binding of specific endocrine FGFs and FGFRs activation [65]. The fifth non-tyrosine kinase FGFR (FGFRL1) provides additional regulation through binding of FGF ligands and potentially functioning as a decoy receptor, an inhibitor of FGFR tyrosine kinase dimerization induction, or a regulator of receptor turnover or signaling [13].

In order to ensure precise and transient signaling, it is essential that pathways possess negative feedback mechanisms to halt signaling or fine-tune activity. Within cells, the RAS-MAPK pathway has the capacity to exert direct negative feedback on FGFRs, where activated ERK1 and ERK2 have been shown to phosphorylate FGFR1 and inhibit its tyrosine kinase activity [66]. Concurrently, numerous specialized inhibitory proteins regulate signaling [67, 68].

Sprouty (SPRY), which functions as an intracellular negative regulator of receptor tyrosine kinases, attenuates FGF/FGFR signaling by interacting with GRB2 and/or RAF [69–72]. Similar expression to FGF (SEF) is a transmembrane protein that has been shown to inhibit the dissociation of the MEK-MAPK (ERK1/2) complex by binding to activated MEK-ERK complex [73, 74]. This results in a blockade of the nuclear translocation of activated MAPKs [75]. The extracellular domain of SEF may also directly interact with FGFR to inhibit receptor phosphorylation [76]. Additionally, dual-specificity phosphatase 6 (DUSP6) encodes an ERK-specific MAPK phosphatase (MKP3) that rapidly terminates signaling by directly dephosphorylating ERK/MAPKs [77]. The regulation of GRB2 in signaling is complex. It functions as a bridging protein, connecting FRS2 to the RAS-MAPK and PI3K-AKT pathways. Additionally, it interacts directly with FGFR2c, contributing to the stabilization of FGFR dimers [78]. Autophosphorylation of GRB2 has been demonstrated to result in its dissociation from the C-terminal region of FGFR, thus enabling full receptor activation [79]. It has been demonstrated that lower levels of GRB2 permit PLCγ binding and enhance phospholipase activity, thereby increasing cell motility [80]. This activity has been shown to promote metastatic behavior in melanoma cells [80]. Conversely, elevated GRB2 levels have been shown to impede PLCγ binding to FGFR [80]. Furthermore, Growth factor receptor bound protein 14 (GRB14) has been shown to function as an inhibitory protein, with the capacity to selectively impede the interaction between PLCγ and FGFR [81]. The tyrosine phosphatase Src homology region 2-containing protein tyrosine phosphatase 2 (SHP2) has been observed to bind to phosphorylated FRS2, a process which has been shown to result in the dephosphorylation of both FGFR2 and GRB2 [78]. Genetic evidence indicates that the formation of the FRS2α-SHP2 complex and its recruitment to the FGF receptor are crucial for downstream ERK signaling, and constitutively activated Ras signaling can rescue the coloboma defect in FGF signaling mutants [82]. Conversely, GRB2 has been observed to impede SHP2 phosphorylation, activation, and FGFR binding (Fig. 2) [79].

The final mechanism of signal termination is receptor ubiquitination and degradation. The ubiquitin ligase casitas B-lineage lymphoma proto-oncogene (CBL) forms a ternary complex with phosphorylated FRS2α and GRB2, which results in the ubiquitination and degradation of FGFR and FRS2α in response to FGF stimulation [83, 84]. Upon the FGFR2 activation, CBL asnd PI3K constitute a complex through the Src homology region 2 and 3 of PI3K and the proline-rich region of CBL. This formation of CBL-PI3K molecular attenuates the PI3K/AKT signaling, leading to increased apoptosis of human osteoblasts (Fig. 2) [83, 84].

## Roles of FGFs in health

**Fig. 4:**
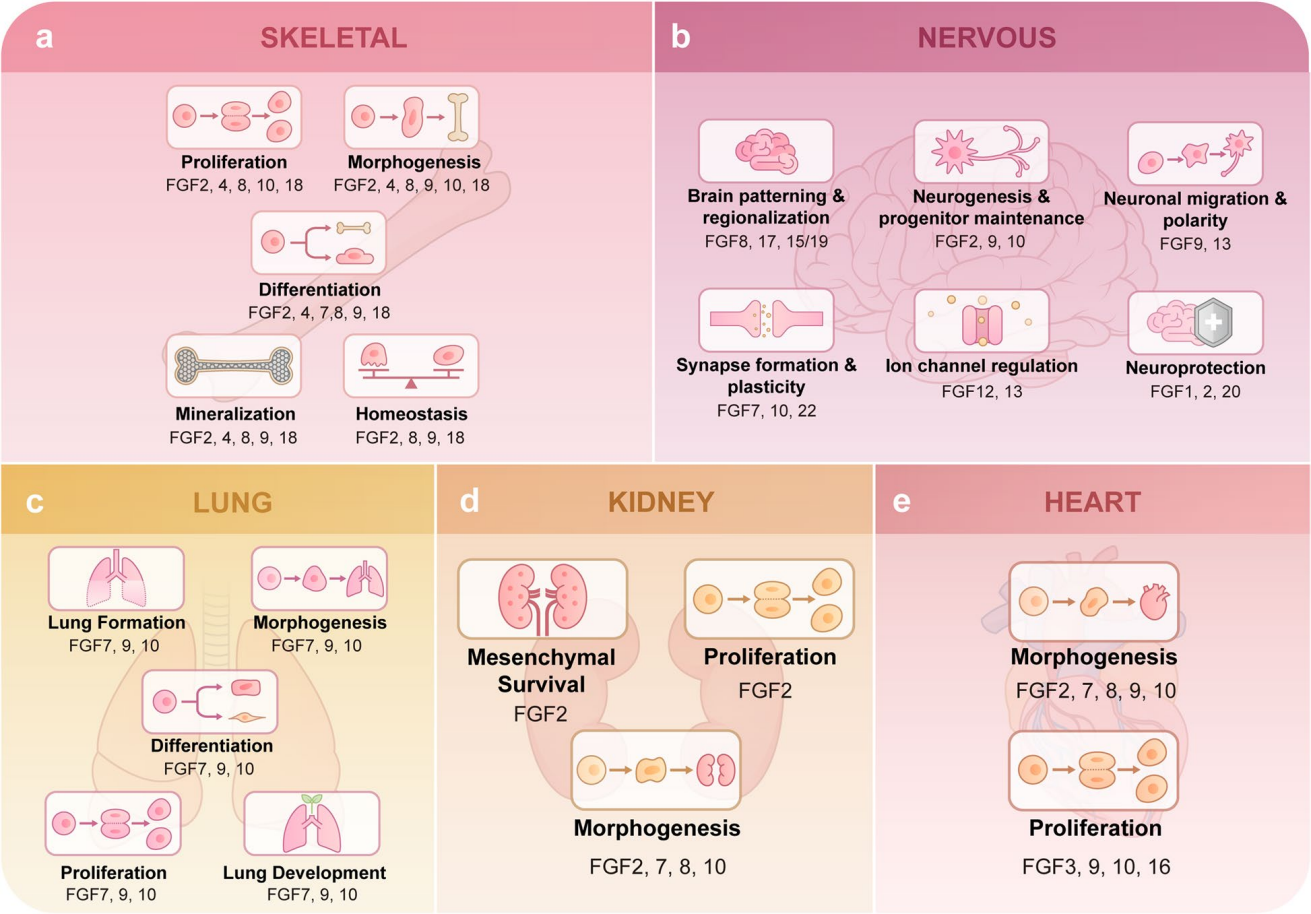
FGF signaling in health. FGF signaling plays a critical role in physiological conditions. **a** Skeletal System. FGF2, FGF4, FGF8, FGF10, and FGF18 collectively regulate the proliferation of skeletal cells. FGF2, FGF4, FGF8, FGF9, FGF10, and FGF18 are crucial for skeletal morphogenesis. FGF2, FGF4, FGF7, FGF8, FGF9, and FGF18 are involved in driving the skeletal differentiation process. FGF2, FGF4, FGF8, FGF9, and FGF18 are associated with bone mineralization. FGF2, FGF8, FGF9, and FGF18 play key roles in maintaining skeletal homeostasis. **b** Brain. FGF8, 17, and 15/19 are associated with brain patterning and regionalization. FGF2, 9, and 10 maintain neurogenesis and progenitor populations. FGF9 and 13 influence neuronal migration and polarity. FGF7, 10, and 22 are involved in synapse formation and plasticity. FGF12 and 13 modulate ion channel function, and FGF1, 2, and 20 contribute to neuroprotection. **c** Lung. FGF7, FGF9, and FGF10 function as master regulators that collectively orchestrate multiple stages of lung development. Their coordinated actions are essential for key processes including initial tissue morphogenesis, epithelial branching, and the subsequent proliferation and differentiation of pulmonary cells. **d** Kidney. FGF2 provides protective and promotional effects on renal mesenchymal cell survival and proliferation. FGF2, FGF7, FGF8, and FGF10 collectively mediate renal morphogenesis. **e** Heart. FGF2, FGF7, FGF8, FGF9, FGF10 and FGF15/19 are instrumental in regulating cardiac morphogenesis. FGF3, FGF9, FGF10, and FGF16 are important factors that promote cardiomyocyte proliferation

FGFs form a highly conserved signaling network that governs organ development and sustains tissue homeostasis under physiological conditions. Through ligand- and receptor-specific interactions, FGF signaling regulates fundamental cellular processes, including progenitor maintenance, lineage commitment, cell survival, and intercellular communication. Although initially characterized as developmental morphogens, many FGF pathways remain active or readily inducible in adult tissues, where they contribute to the maintenance of structural integrity and functional stability. In this section, we focus on the roles of FGFs in health, emphasizing how context-dependent FGF signaling programs established during development are reutilized to support tissue homeostasis and provide a mechanistic basis for adaptive and regenerative responses (Fig. 4).

### Development biology

#### FGF signaling in the skeletal and craniofacial system

FGF signaling plays a fundamental role in skeletal and craniofacial development by coordinating chondrocyte proliferation, osteoblast differentiation, and tissue mineralization. During embryogenesis, distinct FGF ligands regulate both endochondral and intramembranous ossification through tightly controlled spatial and temporal signaling, thereby shaping skeletal patterning and growth. These developmental programs lay the foundation for postnatal bone homeostasis and adaptive remodeling.

FGF2 is broadly expressed in osteoblasts, chondrocytes, and limb bud mesenchyme, where it regulates skeletal morphogenesis [85, 86]. It promotes osteogenic and chondrogenic differentiation of cranial neural crest–derived cells [87], while transgenic overexpression in mice leads to premature growth plate closure, shortened long bones, and reduced mineralization [88, 89]. Interestingly, *Stat1* deletion rescues the chondrodysplasia phenotype, indicating its essential role in FGF2-mediated growth plate regulation [90]. Although *Fgf2* knockout mice show normal limb patterning due to redundancy with FGF4/FGF8, they highlight the context-dependent requirement of FGF2 [91]. Isoform-specific studies reveal that secreted low-molecular-weight FGF2 enhances osteogenesis and bone mass, whereas nuclear high-molecular-weight isoforms suppress mineralization and induce dwarfism with rickets-like features through activation of FGF23/FGFR1/Klotho signaling [92, 93]. These findings establish FGF2 as a dual, isoform-dependent regulator of bone formation and mineralization.

Beyond development, FGF2 is indispensable for skeletal homeostasis. *Fgf2*-null mice gradually develop osteoporosis, emphasizing its importance in adult bone remodeling [94]. FGF2 promotes osteoblast proliferation and differentiation, stimulates bone formation, and enhances mineralization. However, its effects are context-dependent, with continuous treatment inhibiting osteoblast differentiation and intermittent treatment stimulating bone formation [95]. FGF2 also plays a role in fracture repair and can restore bone mass in models of osteoporosis [95]. Research indicates that FGF2 is crucial for the anabolic effects of parathyroid hormone (PTH) on bone. In *Fgf2*^−/−^ mice, the anabolic response to PTH is reduced. PTH increases Runx2 protein expression in wild-type osteoblasts but not in *Fgf2*^−/−^ osteoblasts, and this effect is mediated by cAMP-response element binding protein (CREB) phosphorylation [96].

FGF4 is transiently expressed in the apical ectodermal ridge (AER) during early limb development, where it provides proliferative and morphogenetic signals, though its function can be compensated by other FGFs [97]. In craniofacial bones, FGF4 is expressed in suture mesenchyme and promotes premature suture fusion and mineralization when overexpressed [86]. It also enhances bone marrow stromal cell proliferation and induces *Runx2* expression in osteogenic precursors [86]. Beyond the skeleton, FGF4 is a key epithelial signal regulated by Wnt/LEF1 during early tooth development. Its expression in the dental epithelium initiates epithelial–mesenchymal interactions essential for morphogenesis. Loss of FGF4 leads to developmental arrest of tooth germs, while exogenous FGF4 restores normal development. Acting downstream of Wnt signaling, FGF4 induces *Fgf3* in the mesenchyme and *Shh* in the epithelium, forming a reciprocal signaling loop critical for proper tooth formation [98].

FGF7 plays significant regulatory roles in osteogenic differentiation and intercellular communication. In vitro evidence confirms that exogenous FGF7 specifically promotes the osteogenic differentiation of mouse embryonic stem cells, a process signaled partly through the ERK/Runx2 pathway [99]. In rat bone defect models, recombinant FGF7 notably enhanced bone formation, leading to increased trabecular thickness and upregulated expression of key osteogenic markers, including RUNX2 and BMP2 [100]. Furthermore, it facilitates cell–cell coupling by promoting the expression of Connexin 43 and engaging the MAPK and PI3K-AKT pathways [101]. Despite this clear in vitro role, the lack of a prominent skeletal phenotype in *Fgf7* null mice suggests potential functional compensation by other FGF family members [102].

FGF8 is broadly expressed in the developing limb bud and is indispensable for skeletal morphogenesis [103, 104]. Global deletion of *Fgf8* causes early embryonic lethality [105, 106], while conditional deletion in the limb AER leads to severe limb malformations, including radial and digital defects, underscoring its essential role in bone patterning during limb development [107, 108]. Beyond the limb, FGF8 is expressed in perichondrium, cranial cortical bone, and growth plates, where it influences osteoblast differentiation [109]. In vitro studies show that FGF8 can drive osteogenic differentiation of bone marrow stromal cells and osteoblasts, though some reports suggest inhibitory effects on mineralization, indicating cell type–dependent outcomes [110, 111]. Overall, FGF8 is required for normal skeletal development and contributes to osteogenic lineage regulation. Mechanistically, FGF8 signals through FGFR1 and FGFR3 to activate MAPK–ERK and PI3K–AKT pathways, thereby regulating chondrocyte proliferation and differentiation and contributing to cartilage and bone homeostasis [112].

FGF9, highly expressed in the AER, perichondrium, growth plate chondrocytes, and trabecular bone, is essential for endochondral ossification [85, 86]. Genetic deletion of *Fgf9* results in perinatal lethality due to lung malformations and is associated with proximal skeletal shortening, indicating its requirement after mesenchymal condensation for normal endochondral bone elongation [113, 114]. Conversely, transgenic overexpression of *Fgf9* in chondrocytes (*Col2a1*-*Fgf9*) produces dwarfism, shortened limbs, and vertebral defects, phenotypes resembling FGFR3 activation, caused by reduced chondrocyte proliferation and terminal differentiation [115]. FGF9 deficiency also impairs osteogenesis and decreases osteoclast formation, highlighting its role in coupling bone formation and resorption [113]. In cranial sutures, FGF9 promotes a shift from intramembranous to endochondral ossification, highlighting its capacity to influence mesenchymal fate [116]. FGF9 exerts dual effects on skeletal homeostasis by modulating both osteogenesis and adipogenesis. At the cellular level, FGF9 exerts dual effects on skeletal homeostasis by regulating osteogenesis and adipogenesis. Osteoblast-derived FGF9 is essential for bone balance in male mice, where it enhances osteoprogenitor proliferation but inhibits osteoblast differentiation, likely via a paracrine mechanism involving AKT signaling [117]. In contrast, FGF9 suppresses osteogenic differentiation and mineralization of bone marrow mesenchymal stem cells (BMSCs) mainly through MEK/ERK and partially via PI3K/AKT pathways, while simultaneously promoting osteoclastogenesis by enhancing precursor aggregation and fusion through coordinated activation of MAPK and PI3K/AKT signaling [118]. Beyond bone formation and resorption, FGF9 also shifts BMSCs fate toward adipogenesis at the expense of osteogenesis, particularly during early differentiation. This is supported by *Fgf9* loss-of-function (S99N) mutant mice, which show reduced marrow adiposity and resistance to ovariectomy-induced bone loss. Mechanistically, FGF9 upregulates adipogenic genes while suppressing osteogenic programs via PI3K/AKT/Hippo signaling and MAPK/ERK inhibition of osteogenesis, thereby linking FGF9 activity to the regulation of bone–fat balance [119].

FGF10 plays a pivotal role in limb skeletal development. *Fgf10* knockout mice completely lack forelimbs and hindlimbs and die shortly after birth due to lung agenesis [120, 121]. In these embryos, limb bud initiation occurs but subsequent outgrowth is severely impaired, indicating that FGF10 is indispensable for sustained limb bud proliferation and skeletal element formation, although clavicle development remains unaffected [120, 121]. While its essential contribution to embryonic limb morphogenesis is well established, the role of FGF10 in postnatal bone growth and remodeling remains unclear. FGFs secreted from the AER, most notably FGF4 and FGF8, provide key signals that activate FGFR1 and FGFR2 in the underlying mesenchyme, thereby initiating *Sox9* expression and priming progenitors for chondrogenic differentiation [122]. In parallel, mesoderm-derived FGF10 initiates AER formation via FGFR2b, while AER-derived FGF8 sustains mesenchymal FGF10 expression, forming a reciprocal FGF10–FGF8 feedback loop [123, 124]. This circuit maintains mesenchymal proliferation and AER integrity, ensuring continuous limb outgrowth and proper proximodistal skeletal patterning.

FGF18 is expressed in osteogenic mesenchyme, differentiating osteoblasts of the calvaria, the perichondrium, joints, and growth plates [85, 86]. *Fgf18* knockout mice die shortly after birth, showing expanded chondrocyte proliferation and hypertrophy similar to *Fgfr3* deficiency, indicating that FGF18 restricts chondrogenesis during endochondral ossification [125–127]. In contrast, in vitro studies demonstrate that FGF18 can stimulate proliferation and differentiation of primary chondrocytes and ATDC5 cells, as well as enhance early chondrogenesis by potentiating bone morphogenetic protein (BMP) activity through suppression of noggin [128, 129]. These findings indicate that the role of FGF18 in cartilage formation is stage- and context-dependent. In bone formation, *Fgf18* knockout mice exhibit delayed suture closure, reduced osteoblast proliferation and differentiation, and impaired mineralization in cranial and long bones [125–127]. FGF18 also promotes osteoblast proliferation in vitro and regulates skeletal vascularization and osteoclast recruitment [127, 128]. These findings identify FGF18 as a key regulator of both intramembranous and endochondral ossification through coordinated effects on chondrocytes, osteoblasts, and bone vasculature. FGF18 plays a pivotal role in bone homeostasis through its regulation of autophagy in chondrocytes. Research has shown that FGF18 promotes chondrocyte autophagy via FGFR4 and JNK activation. This autophagy induction facilitates type II collagen secretion and maintains extracellular matrix (ECM) homeostasis. FGF18 deficiency results in autophagy suppression, collagen accumulation in the endoplasmic reticulum (ER), and impaired bone growth [130].

#### FGF signaling in the nervous system

FGF signaling is essential for nervous system development, governing brain regionalization, neural progenitor maintenance, and neuronal differentiation. During early embryogenesis, multiple FGFs establish morphogen gradients that define major brain axes and segmental identities, while later supporting neuronal migration, synapse formation, and circuit assembly. These early patterning and differentiation cues contribute to long-term neural stability and functional homeostasis.

As the two earliest discovered FGF molecules, FGF1 and FGF2 play certain roles in maintaining nervous system homeostasis. FGF1 is widely distributed throughout the central nervous system (CNS) during both embryonic and adult stages. In the adult human brain, its expression peaks in oligodendrocytes and astrocytes, rendering it indispensable for the maintenance of nervous system homeostasis. Homozygous mice deficient in FGF1 appear phenotypically normal and fertile under standard housing conditions; however, they demonstrate heightened sensitivity to external stimuli. Under high-fat dietary conditions, mutant mice exhibit impaired spatial memory and depression-like behaviors [131]. FGF2 is highly expressed commencing from the neurulation stage and influences the development of the hippocampus and substantia nigra [132]. During adulthood, FGF2 is expressed in neurogenic niches—the subventricular zone (SVZ) of the lateral ventricles and the subgranular zone (SGZ) of the hippocampal dentate gyrus—and participates in the regulation of adult neurogenesis through modulation of proliferation and differentiation of adult neural stem and progenitor cells [133].

FGF3 expression is highly concentrated during the embryonic period, with only minimal maintenance in the cerebellum postnatally. It is primarily associated with the development of the vestibulocochlear ganglion; mutations in FGF3 can lead to developmental defects in the central auditory-vestibular pathway (citation missing). Similar to FGF-3, the FGF-6, FGF-7, and FGF-8 genes demonstrate higher expression levels during embryonic stages compared to postnatal stages, indicating their involvement in brain development. Among these, FGF-6 expression in perinatal mice is restricted to the central nervous system and skeletal muscle; however, FGF-6 knockout mice remain viable and fertile without observable abnormal phenotypes [134]. Biallelic knockout mouse models of FGF7 are also viable, yet mice lacking FGF7 expression (FGF7-KO) exhibit hippocampal epileptogenic alterations accompanied by remarkably enhanced neurogenesis and mossy fiber sprouting [135]. Furthermore, FGF7 has been reported to participate in nerve injury repair and injury-induced nociception in the peripheral nervous system, demonstrating upregulation in damaged nerves. FGF7-KO mouse models also display acute inflammatory nociceptive responses, indicating that FGF7 plays a significant role in maintaining nervous system stability [136]. FGF8 serves as a principal regulator of brain regional patterning and development. It is critically important for establishing the midbrain-hindbrain boundary (the isthmic organizer region) during early developmental processes. FGF8 diffusion gradients control patterning in posterior brain regions [137]. In vitro, FGF8 can also promote cellular heterogeneity in human induced pluripotent stem cells (hiPSCs), leading to the formation of multi-regional organoids (telencephalic and mesencephalic-like domains) [138].

FGF9 was originally identified during screening for cytokines acting upon the central nervous system [139]. Secreted by neurons, FGF9 is widely expressed during embryonic development [140]. In adult rats, FGF9 is expressed in multiple regions including the cerebral cortex, cerebellum, hypothalamus, and brainstem, and is essential for granule neuron migration and the formation of Bergmann glial cell radial fiber scaffolds. Loss of FGF9 affects the timing of astrocyte maturation and synaptic structure formation. FGF9 deficiency in both central and peripheral nervous systems leads to cerebellar developmental abnormalities, manifested as ataxia and motor coordination disorders [141], accompanied by epileptic phenotypes. Recent studies have additionally demonstrated that somatosensory nerves can regulate periosteal cell proliferation and differentiation through FGF9 signaling to promote fracture healing [142]. In contrast to the broad role of FGF9, FGF10 is associated with forebrain development. It is expressed in the embryonic forebrain and regulates the transition of neural stem/progenitor cells to radial glial cells, thereby influencing the generation of neurons and basal progenitors [143]. FGF10 continues to be expressed in the adult mouse brain, including in the hippocampus, hypothalamus, and cerebellum, and is localized adjacent to neural stem cell niches, suggesting its potential role in neural homeostasis and regeneration [144].

FGF12, FGF13, and FGF15/19 are all widely distributed throughout the nervous system during the embryonic period. FGF12 can interact with sodium channels in the nervous system and is highly expressed in neurons [145]. It is widely distributed during early embryonic stages, subsequently becoming gradually restricted to the retinal ganglion cell layer, inner ear spiral ganglion, and vestibular ganglion, while simultaneously entering peripheral somatosensory neurons [146]. Postnatally, FGF12 protein is enriched in pyramidal cells of the cerebral cortex, hippocampal regions, cerebellar granule cells, and spinal motor neurons [147]. Its mutations can lead to epileptic encephalopathy [148], cerebellar atrophy, hearing abnormalities, and balance disorders [149]. FGF13 is essential for neural development and is continuously expressed in the mouse central nervous system (including the cerebral cortex, hippocampus, and spinal cord) and peripheral nervous system (dorsal root ganglia, cranial ganglia, and enteric nervous system) from embryonic stages through adulthood. During cerebral cortex development, it regulates the establishment of neuronal polarity and maintains growth cone microtubule stability [150]. FGF13 deficiency or mutation leads to migration disorders, epileptiform discharges, and learning and memory deficits in mice [151]. FGF15 and FGF19 are orthologs across different species and the mouse *Fgf15* gene was originally discovered in the embryonic mouse central nervous system [152]. Subsequently, its human ortholog FGF19 was identified in embryonic and fetal human brain tissues [153, 154]. FGF15/19 plays extremely critical roles in central nervous system development. It is expressed throughout the developing central nervous system, primarily functioning as a regionalization and pro-differentiation signal in the embryonic nervous system. Acting as a downstream effector of master signals such as Shh and FGF8, it constrains the proliferation/differentiation balance of neuronal precursors in different segments of the forebrain, midbrain, diencephalon, and hindbrain. *Fgf15*^*−/−*^ mice exhibit tectal hypoplasia, thalamocortical connection disorders, and increased seizure susceptibility, suggesting that early signal imbalance may lead to long-term neural circuit pathologies [155].

Similar to FGF8, FGF17 is tightly localized to specific regions of the developing brain and is expressed only during early stages of proliferation and neurogenesis in embryos [156]. FGF17 is mainly distributed at the midbrain-hindbrain junction and dorsal forebrain during the embryonic period, cooperating with FGF8 to constitute the midbrain-hindbrain boundary signaling gradient. However, postnatally, except for small amounts retained in the cerebellar vermis and inferior colliculus, overall expression is rapidly downregulated, suggesting that its primary functional window is the embryonic-neonatal period. *Fgf17*⁻/⁻ mice show no morphological defects in the cerebellar hemispheres at postnatal day 2 and at 2–3 months of age, yet the inferior colliculus and vermis cerebellum are smaller [157]. This may be related to the premature differentiation of Purkinje cells observed in *Fgf17*⁻/⁻ mice at embryonic day 14. Furthermore, *Fgf17* has been found to participate in hippocampal oligodendrocyte proliferation and differentiation, contributing to long-term memory performance in mice [158]. Patients with human 8p21.3 microdeletions (including *FGF17*) present with Dandy-Walker malformation, vermis hypoplasia or dysplasia, and expansion of the fourth ventricle and posterior cranial fossa cistern [159].

FGF20 is a neurotrophic factor belonging to the FGF9 subfamily [160]. Among 39 human tissue types, FGF20 was found to be highly expressed in the cerebellum and demonstrated lower expression in the whole brain, hippocampus, substantia nigra, and spinal cord—indicating its important role in the brain [161]. Both exogenous and endogenous FGF20 provide neuroprotective effects to dopaminergic neurons by preventing their loss in the substantia nigra pars compacta and preventing motor impairments [162, 163]. FGF22 is also closely related to brain development. In zebrafish models, FGF22 is expressed in the forebrain, midbrain, and midbrain-hindbrain boundary regions, *Fgf22* knockout leads to forebrain structural abnormalities, blocked differentiation of glutamatergic neurons and GABAergic interneurons, suppressed astrocyte development, and enhanced oligodendrocyte generation. It is also critically important for synapse formation. Knockout of *Fgf22* in mice results in reduced synaptic vesicle clustering, smaller presynaptic structure volume, and decreased synaptic transmission efficiency [164]. FGF22 also exhibits synergistic effects with other FGF factors, together with FGF7 and FGF10, FGF22 acts as a presynaptic organizer factor in the brain, promoting differentiation of axon terminals and participating in synapse formation and functional maintenance [165].

#### FGF signaling in the lung development

FGF signaling is a central driver of lung development, directing the initiation of lung buds, branching morphogenesis, and distal epithelial fate specification. Through dynamic interactions between mesenchymal and epithelial compartments, distinct FGF ligands coordinate airway patterning and alveolar formation in a stage-dependent manner. These developmental signaling circuits are subsequently reutilized during postnatal lung maintenance and repair.

FGF7 is critically involved in the self-renewal of alveolar type II (AT2) cells and the promotion of alveolar growth [166]. In addition, it can induce the formation of alveolus-like organoids from embryonic lung epithelium in culture, indicating a complementary role in driving distal epithelial differentiation [167]. Although several studies suggest a contribution of FGF7 to normal lung morphogenesis, *Fgf7* knockout mice display normal lung histology and survive well, indicating that its function in lung development may be redundant or compensated by other FGFs [102]. The precise origin of pulmonary FGF7 expression and its direct relationship with AT2 cell function in vivo remain to be clarified.

FGF9 is a key regulator of lung morphogenesis with diverse functions across multiple compartments. In the distal mesenchyme, it prevents premature smooth muscle cell (SMC) differentiation, thereby ensuring proper airway SMC organization [168]. At the same time, mesothelium- and epithelium-derived FGF9 promotes proliferation of both epithelial and mesenchymal cells, in part by positively regulating *Fgf10* expression, and also contributes to pulmonary vascular development [169, 170]. *Fgf9* knockout mice die at birth from lung hypoplasia with reduced mesenchymal growth and impaired epithelial branching [141]. Mechanistically, FGF9 acts through FGFR3 in epithelial cells to promote distal epithelial identity and restrain premature differentiation, primarily via the PI3K pathway. These functions are counterbalanced by FGF10–FGFR2b signaling, which enhances epithelial proliferation and differentiation through MAPK activation, highlighting the coordinated yet antagonistic interplay of FGF9 and FGF10 in sculpting the developing lung epithelium [171].

FGF10 is indispensable for lung development, acting through mesenchymal expression and signaling via FGFR2b in epithelial cells to initiate and sustain lung bud outgrowth [172]. Its expression remains stable during the pseudoglandular stage and increases markedly in the canalicular stage, where it supports distal fate specification and airway epithelial differentiation [173]. In situ hybridization studies show diffuse expression of FGF10 throughout the human lung parenchyma, including airway and vascular smooth muscle cells, suggesting roles beyond branching morphogenesis [174]. Genetic studies link rare *FGF10* mutations or SNPs to acinar dysplasia and alveolar development disorders, typically manifesting after the pseudoglandular stage. Conversely, excess FGF10 disrupts branching and causes severe dysplasia due to uncontrolled epithelial proliferation [175]. Together, these findings underscore FGF10 as a central regulator of branching, distal epithelial identity, and alveolar formation during lung morphogenesis.

#### FGF signaling in the urinary system

FGF signaling governs kidney development by regulating mesenchymal survival, nephron progenitor maintenance, and branching morphogenesis of the collecting duct system. During nephrogenesis, multiple FGFs act in a coordinated yet non-redundant manner to ensure proper nephron formation and renal architecture. This developmental dependence on FGF signaling underscores its importance in maintaining renal structural and functional integrity.

Early studies showed that exogenous FGF2 can sustain metanephric mesenchyme (MM) survival and proliferation, and in some cases induce epithelial nephron structures in explant culture, indicating its supportive role at the onset of nephron formation [176, 177]. Genetic evidence further established the essential function of FGF8: conditional deletion of *Fgf8* in the mesenchyme or intermediate mesoderm arrests nephron development beyond the renal vesicle stage, leading to severely hypoplastic kidneys and complete failure of nephron formation [178, 179].

In parallel, FGF9 and FGF20 are indispensable for maintaining the nephron progenitor pool. Double knockout mice exhibit extensive progenitor apoptosis and kidney hypoplasia, while exogenous FGF9 or FGF20 preserves MM and isolated progenitors in vitro [180]. By comparison, knockout of *Fgf1* or *Fgf2*, alone or in combination, does not result in apparent renal defects, suggesting that they are dispensable or functionally compensated during kidney development [181].

Other FGFs primarily regulate the collecting duct system. *Fgf7* knockout mice display markedly reduced ureteric bud branching and collecting duct maturation, accompanied by secondary nephron loss [182]. Exogenous FGF7 promotes ureteric bud outgrowth and increases nephron numbers in culture [182]. Similarly, *Fgf10* deficiency results in smaller kidneys with fewer collecting ducts, underscoring its role in branching morphogenesis [121].

#### FGF signaling in the heart development

FGF signaling is indispensable for heart development, contributing to second heart field expansion (SHF), outflow tract formation (OFT), and myocardial growth. Distinct FGF ligands function in a region-specific and stage-dependent manner to regulate cardiomyocyte proliferation and cardiac morphogenesis. These developmental roles establish the structural and signaling basis for normal cardiac function.

FGF8 is expressed during early embryogenesis and is required for cardiac looping, anterior heart field development, and the survival of migratory cardiac neural crest cells; its loss results in OFT septation defects [183, 184]. FGF9, expressed in the embryonic heart, functions as a paracrine growth factor for cardiomyocytes, and knockout mice display reduced myocardial proliferation and smaller embryonic hearts, along with ventricular hypoplasia and thinner ventricular walls [185]. FGF10 regulates region-specific cardiomyocyte proliferation, particularly in the right ventricle, and its deficiency leads to ventricular hypoplasia, impaired epicardial-derived cell migration, and thin-walled hearts [186, 187]. Together with FGF3, it also supports cardiovascular progenitor development and overlaps functionally with FGF8 in SHF-derived structures such as the OFT and right ventricle [188, 189].

Additional FGFs provide complementary roles. FGF15/19, expressed in the pharyngeal arches, is essential for OFT morphogenesis, as knockout mice exhibit great artery misalignment linked to early OFT defects [190]. FGF16, expressed in embryonic cardiomyocytes, promotes myocardial proliferation and growth; its deletion reduces heart weight and cardiomyocyte number, resembling the phenotype of *Fgf9* deficiency [191]. Notably, genetic background influences the severity of *Fgf16* loss, with some strains displaying embryonic lethality accompanied by chamber dilation and wall thinning [192, 193].

### Tissue repair and homeostasis

FGFs comprise a conserved family of signaling proteins that play essential roles in tissue repair and the maintenance of tissue homeostasis. In adult tissues, FGFs and their receptors are widely expressed and maintained in a readily activatable state, allowing rapid responses to tissue stress or injury [194, 195]. Upon damage, FGF signaling is promptly enhanced through increased ligand availability and receptor activation, positioning FGFs as key regulators that couple injury sensing to coordinated cellular responses [194, 195]. Importantly, FGF activity is not restricted to regenerative phases but extends into post repair remodeling, where it contributes to the restoration of structural integrity and functional homeostasis.

FGF1, traditionally characterized as a mitogenic growth factor, is increasingly recognized as a pleiotropic regulator of tissue repair and organ protection. In acute inflammatory settings, FGF1 attenuates tissue damage, edema, and inflammatory cell infiltration by inhibiting TLR4–NF-κB signaling and enhancing Nrf2-dependent antioxidant defenses, thereby limiting apoptosis and promoting tissue preservation [196]. Beyond inflammation control, FGF1 also plays a critical role in metabolic and epithelial repair. In drug-induced liver injury, hepatic FGF1 acts as an endogenous safeguard against bile acid–mediated hepatotoxicity by activating FGFR4–ERK1/2 signaling to repress HNF4α-driven bile acid synthesis and restore metabolic homeostasis [197]. Similarly, in intestinal injury, non-mitogenic FGF1 variants suppress MAPK-dependent chemokine expression in epithelial cells, reducing neutrophil recruitment and mucosal inflammation [198]. In chronic metabolic disorders such as diabetic cardiomyopathy, FGF1 further preserves tissue function by improving mitochondrial integrity and energy metabolism through AMPK–Nur77 signaling [199], collectively highlighting FGF1 as a central integrator of anti-inflammatory, antioxidant, and metabolic programs that support tissue repair and functional recovery.

FGF2 is a central mediator of tissue repair that coordinates epithelial regeneration, vascular protection, and neural recovery across inflammatory and ischemic injuries. In barrier tissues, FGF2 cooperates with inflammatory cues to promote epithelial proliferation and repair, exemplified by its synergistic action with IL-17 in restoring intestinal epithelial integrity after damage through controlled ERK signaling crosstalk [200]. In parenchymal organs, FGF2 supports regenerative homeostasis by enhancing antioxidative and proliferative programs, such as activating the USP42–PPARγ axis during liver regeneration to counteract toxin-induced oxidative injury [201]. In ischemia–reperfusion settings, FGF2 preserves microvascular function and tissue viability by protecting endothelial cells from oxidative stress and ferroptosis via AMPK–HDAC5–KLF2–dependent pathways [202]. Beyond peripheral tissues, FGF2 also exerts potent neuroprotective and neuroregenerative effects by sustaining ERK-dependent neurogenesis, modulating glial responses, and promoting functional recovery following neuroinflammation or spinal cord injury [203, 204].

FGF4 functions as a stress-responsive repair factor that protects tissue integrity by suppressing apoptosis, restoring metabolic homeostasis, and limiting injury-associated inflammation across multiple organs. In the liver, FGF4 is dynamically induced during immune-mediated, metabolic, and toxic injuries, where it acts predominantly through FGFR4 to preserve hepatocyte survival, restrain oxidative stress, and normalize lipid metabolism via Ca^2^⁺–CaMKKβ–AMPK–dependent signaling cascades [205, 206]. Loss of hepatic FGF4 exacerbates hepatocellular apoptosis, immune dysregulation, steatosis, and fibrogenesis, whereas recombinant FGF4 mitigates liver injury by enhancing mitochondrial integrity and repressing pro-apoptotic and stress-amplifying pathways [207]. Beyond hepatic repair, FGF4 confers protection in ischemic and chemotherapeutic cardiac injury by activating AMPK signaling to inhibit ferroptosis and preserve myocardial viability [208]. In barrier and filtration tissues, paracrine FGF4 promotes cutaneous wound re-epithelialization through p38 MAPK–dependent keratinocyte migration and safeguards podocyte survival in diabetic kidney disease via FGFR1–AMPK–FOXO1 signaling [209, 210].

FGF7 is a prototypical paracrine repair factor that promotes tissue regeneration while actively restraining fibrotic remodeling across multiple organs. In mesenchymal-rich tissues, FGF7 preserves tissue architecture and function by directing progenitor cell fate decisions, as exemplified in tendons where FGF7 drives tenogenic differentiation while suppressing pro-fibrotic lineage commitment, thereby reducing matrix disorganization and restoring mechanical integrity [211]. In stress-exposed parenchymal cells, FGF7 enhances cell survival and regenerative capacity by mitigating oxidative damage, maintaining mitochondrial homeostasis, and fine-tuning MAPK signaling to favor pro-survival pathways [212]. In the injured liver, FGF7 functions as a key niche-derived signal that activates facultative liver progenitor cells and supports hepatocyte survival and proliferation via FGFR2b-dependent AKT and ERK signaling, leading to attenuation of inflammation and early fibrogenesis through paracrine crosstalk with macrophages and hepatic stellate cells [213, 214].

FGF10 is a paracrine regenerative factor that promotes tissue repair by simultaneously enhancing progenitor cell–driven renewal and limiting maladaptive fibrotic remodeling. In the heart, FGF10 is rapidly induced after injury and supports functional recovery by stimulating cardiomyocyte proliferation while restraining myofibroblast activation, thereby preserving cardiac structure and function through coordinated regulation of Hippo signaling, cell-cycle control, and metabolic reprogramming [215]. In the lung, FGF10 acts as a critical determinant of epithelial cell fate during injury repair, biasing bronchial epithelial stem cells toward alveolar regeneration rather than fibrotic honeycomb remodeling via FGFR2b signaling [216]. In ischemic and oxidative stress–prone tissues, including the liver and skin, FGF10 protects parenchymal and epithelial cells from apoptosis and proliferation arrest by activating PI3K–AKT–NRF2 and ERK–YAP pathways, respectively, promoting both acute cytoprotection and subsequent regenerative responses [217, 218]. Beyond peripheral organs, FGF10 also exerts neuroprotective effects, mitigating neuronal loss and synaptic dysfunction through FGFR2-dependent survival signaling [219].

FGF18 acts as a stress-responsive cytoprotective factor that preserves tissue homeostasis by limiting oxidative damage, inflammation, and fibrosis following injury. In the heart, FGF18 restrains pressure overload–induced pathological hypertrophy by maintaining redox balance, suppressing cardiomyocyte apoptosis, and attenuating fibrotic remodeling through FGFR3-mediated activation of FYN and subsequent inhibition of NOX4-dependent reactive oxygen species production [220]. In the liver, FGF18 is secreted by hepatic stellate cells and protects hepatocytes from ischemia–reperfusion injury by activating the USP16–KEAP1–Nrf2 axis, thereby enhancing antioxidant defenses and cell survival [221]. Consistently, in acute lung injury, FGF18 dampens endothelial inflammation and vascular leakage by suppressing NF-κB signaling, leading to reduced cytokine production and improved barrier integrity [222].

### Metabolic regulation

Endocrine FGFs, particularly FGF15/19, FGF21, and FGF23, have emerged as central regulators of systemic metabolic homeostasis. Unlike paracrine FGFs, these ligands act hormonally through circulation and signal via FGFR–Klotho receptor complexes in distant tissues.

FGF15/19 acts as a gut hormone that controls the synthesis of bile acids [223], modulates hepatic protein and glycogen metabolism [224], and suppresses gluconeogenesis [225]. It is expressed in the small intestine under the transcriptional control of the bile acid-activated farnesoid X receptor (FXR) [226]. FGF15/19 regulates bile acid synthesis by binding to the FGFR4–β-Klotho complex in the liver, inhibiting the expression of the rate-limiting enzyme for bile acid formation, CYP7A1. This negative feedback loop helps maintain bile acid homeostasis [227]. Additionally, FGF15/19 promotes glycogen storage [224] and inhibits gluconeogenesis by suppressing cAMP–CREB signaling pathways or downregulating AKT-mediated FOXO1 dephosphorylation [225, 228].

FGF21 is a hormone primarily produced by the liver and functions as an endocrine messenger to regulate metabolic homeostasis [229]. One of the key functions of FGF21 is its ability to enhance insulin sensitivity [230]. FGF21 has been shown to potentiate insulin action, particularly in adipose tissue, leading to increased glucose uptake and utilization [230, 231]. This effect is particularly pronounced in states of obesity and insulin resistance [230, 231]. FGF21 also plays a significant role in regulating energy expenditure [232]. Chronic administration of FGF21 or its analogues increases energy expenditure and promotes weight loss in animal models and humans [232]. This effect is mediated through CNS actions, particularly in the hypothalamus, where FGF21 signaling increases the activity of glucose-excited neurons, thereby enhancing energy expenditure and reducing body weight [233]. FGF21 has also been implicated in the regulation of hepatic lipid metabolism. It reduces hepatic triglyceride levels by promoting lipid catabolism and reducing lipogenesis. Clinical studies have shown that FGF21 analogues can significantly decrease liver fat and improve liver fibrosis in patients with non-alcoholic steatohepatitis (NASH) [234]. Additionally, FGF21 regulates macronutrient preference. It suppresses carbohydrate intake and sweet taste preference by signaling to the CNS, particularly in response to high carbohydrate intake. This negative feedback loop helps maintain carbohydrate homeostasis [235].

FGF23 is a hormone predominantly produced by osteoblasts and osteocytes, playing a crucial role in maintaining phosphate and vitamin D homeostasis [236]. FGF23 acts as a counter-regulatory hormone to protect the organism from the toxic effects of excess vitamin D and phosphate. It inhibits renal tubular phosphate reabsorption and suppresses the production of 1,25(OH)_2_D, thereby regulating mineral metabolism [237, 238]. FGF23 has emerged as a key player in the bone-kidney axis, coordinating phosphate handling by the kidneys with bone mineralization and turnover. The discovery of FGF23 has expanded our understanding of mineral metabolism by revealing complex interactions between the parathyroid gland, intestines, bone, and kidney [237, 238]. This hormonal network is essential for maintaining systemic mineral homeostasis and energy metabolism.

## Dysregulation of FGFs in disease

**Fig. 5:**
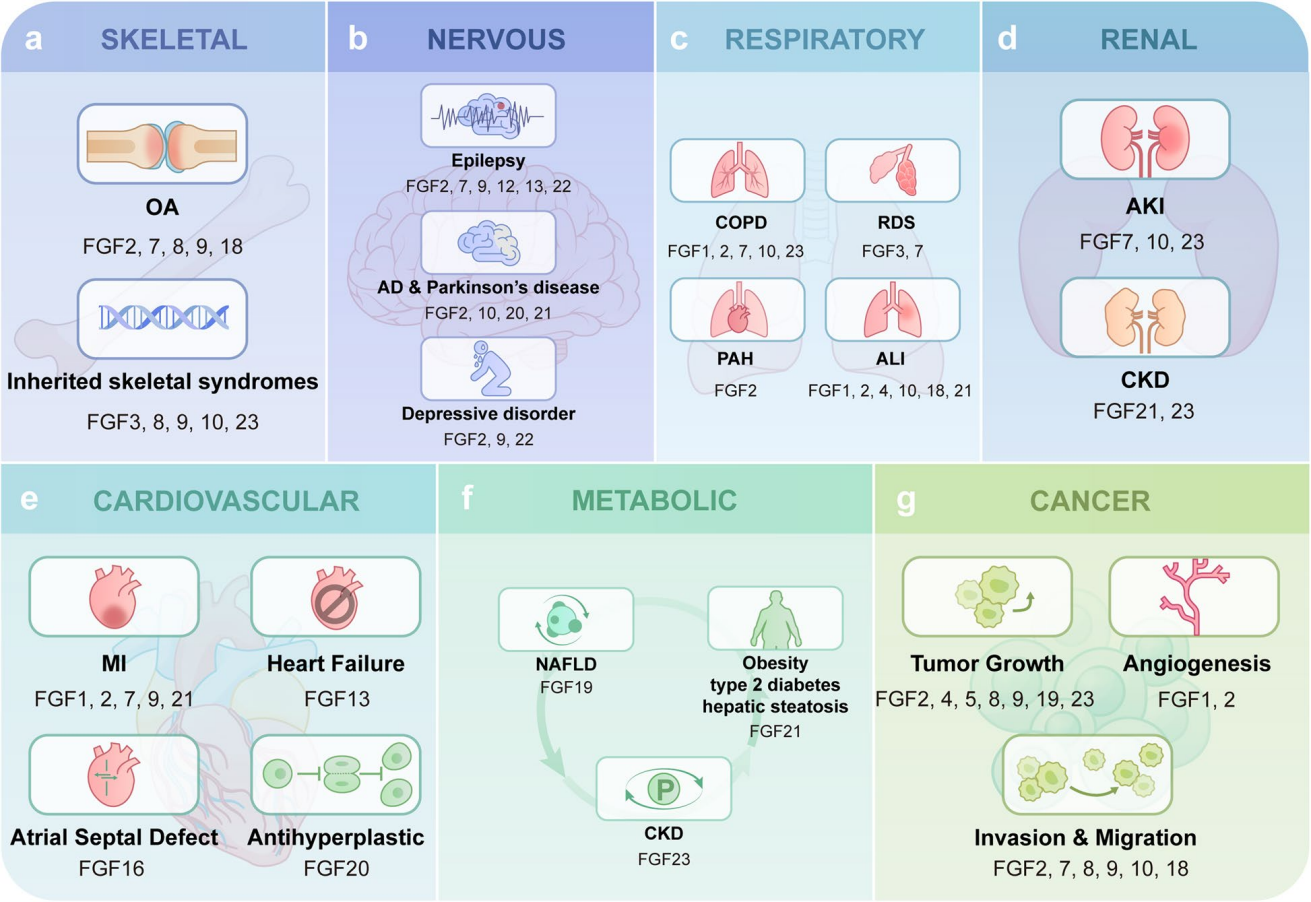
FGF signaling in disease. Dysfunctional FGF signaling is involved in numerous pathological processes. **a** Skeletal System**.** Specific FGFs are implicated in osteoarthritis (OA; FGF2, 7, 8, 9, 18) and inherited skeletal syndromes (FGF3, 8, 9, 10, 23). **b** Nervous System. FGF dysregulation contributes to neurological disorders: epilepsy (FGF2, 7, 9, 12, 13, 22), Alzheimer's and Parkinson's diseases (AD and PD; FGF2, 10, 20, 21), and depressive disorder (FGF2, 9, 22). **c** Respiratory System. FGFs are involved in multiple respiratory diseases, including chronic obstructive pulmonary disease (COPD; FGF1, 2, 7, 10, 23), pulmonary arterial hypertension (PAH; FGF2), cold/airway inflammation (FGF1, 2, 4, 10, 18, 21), and acute lung injury (ALI; FGF1, 2, 4, 10, 18, 21, 23). **d** Renal Disorders. FGFs play roles in acute kidney injury (AKI; FGF7, 10, 23) and chronic kidney disease (CKD; FGF21, 23). **e** Cardiovascular Disorders. FGF signaling contributes to cardiovascular pathologies, including myocardial infarction (MI; FGF1, 2, 7, 9, 21), heart failure (FGF13), atrial septal defect (FGF16), and an antihypertrophic role of FGF20. **f** Metabolic Disorders. FGF19 is associated with non-alcoholic fatty liver disease (NAFLD), while FGF21 is linked to obesity, type 2 diabetes, and hepatic steatosis. FGF23 is also involved in chronic kidney disease (CKD). **g** Cancer. The FGF/FGFR axis drives multiple aspects of cancer progression, including tumor growth (FGF2, 4, 5, 8, 9, 19, 23), angiogenesis (FGF1, 2), and invasion and migration (FGF2, 7, 8, 9, 10, 18)

Dysregulation of FGF signaling is increasingly recognized as a central driver of human disease, reflecting the multifaceted roles of FGFs in development, tissue repair, and metabolic homeostasis. Genetic mutations or aberrant expression of FGFs and their receptors disrupt cellular proliferation, differentiation, and survival, contributing to congenital malformations, degenerative disorders, and chronic organ dysfunction. In skeletal tissues, FGF alterations underlie both inherited syndromes and osteoarthritis progression; in the nervous system, they modulate neurogenesis, excitability, and neuroprotection, impacting epilepsy, neurodegeneration, and mood disorders. Dysregulated FGFs also affect pulmonary and renal homeostasis, cardiovascular adaptation, and systemic metabolism, while aberrant FGF/FGFR activity drives tumor growth, invasion, and angiogenesis. Collectively, these observations highlight FGFs as critical regulators of physiological and pathological processes, offering promising avenues for diagnostic and therapeutic intervention across diverse diseases (Fig. 5).

### Skeletal and craniofacial disorders

In the skeletal and craniofacial system, FGF signaling orchestrates both developmental patterning and adult tissue maintenance. Genetic mutations in FGF ligands and receptors disrupt bone formation, suture fusion, and limb morphogenesis, giving rise to congenital syndromes, while aberrant FGF activity in adulthood regulates cartilage homeostasis, osteophyte formation, and extracellular matrix turnover, driving degenerative diseases such as osteoarthritis. The context- and isoform-dependent effects of FGFs underscore their dual anabolic and catabolic roles, highlighting therapeutic opportunities for skeletal repair and regeneration.

Genetic mutations in FGFs have been directly linked to congenital skeletal disorders. *FGF3* heterozygous mutations lead to syndromes involving deafness, inner ear agenesis, and brachydactyly [239]. *FGF8* mutations contribute to autosomal dominant hypogonadotropic hypogonadism, occasionally accompanied by skeletal abnormalities such as short stature, joint hyperlaxity, clinodactyly, and mild scoliosis [240]. Missense mutations in *FGF9* (S99N and R62G) have been linked to multiple synostoses syndrome type 3, where patients may also exhibit sagittal suture fusion and humeroradial synostoses [241, 242]. *FGF10* loss-of-function variants cause lacrimo-auriculo-dento-digital (LADD) syndrome, characterized by nasolacrimal duct aplasia, ear anomalies, hearing loss, and distal limb malformations [243], and have also been identified as risk factors for nonsyndromic cleft lip with or without cleft palate [244]. Missense variants in *FGF23* (R176Q, R179W, R179Q) are responsible for autosomal dominant hypophosphatemic rickets (ADHR), a condition commonly associated with rickets, skeletal pain, and dental abscesses [245]. In contrast, loss-of-function mutations in *FGF23* lead to hyperphosphatemic familial tumoral calcinosis, a rare autosomal recessive disorder characterized by progressive ectopic calcification and increased serum phosphate levels [246].

FGFs are also critical in acquired skeletal disorders such as osteoarthritis (OA). Multiple FGFs, including FGF2, FGF7, FGF8, FGF9, and FGF18, are involved in cartilage homeostasis and degeneration [247].

FGF2 plays a complex, context-dependent role in the pathogenesis and progression of OA, exhibiting both catabolic and anabolic effects on articular cartilage. On one hand, FGF2 increases the transcription of MMP1 and MMP13, two critical collagenases implicated in cartilage degeneration in human articular chondrocytes [248, 249]. On the other hand, FGF2 reduces IL-1–induced ADAMTS4 and ADAMTS5 activity in human articular cartilage [250]. Furthermore, *Fgf2*-deficient mouse models exhibit accelerated OA progression, whereas exogenous FGF2 administration can rescue these defects [251]. The opposing roles of FGF2 may relate to differences in isoform activity, as well as the dosage, timing, and duration of FGF2 exposure. For example, the functional duality of FGF2 signaling in OA can be explained by the antagonistic roles of its distinct molecular weight isoforms. Genetic studies indicate that ablation of the low molecular weight FGF2 isoform, a secreted autocrine/paracrine factor, exacerbates both spontaneous and mechanically induced OA in mice. This phenotype aligns with a catabolic shift in joint homeostasis, characterized by upregulation of matrix-degrading enzymes such as MMP13 and ADAMTS5, increased expression and activation of the pro-catabolic receptor FGFR1, and activation of the downstream ERK/MAPK pathway. In contrast, mice specifically deficient in high molecular weight FGF2 isoforms, which contain nuclear localization sequences and function primarily intracellularly, are fully protected from OA even under mechanical stress or aging. This protection correlates with an enhanced anabolic environment, notably elevated expression of FGFR3, a receptor known to promote cartilage matrix synthesis and homeostasis. These findings suggest that low molecular weight FGF2 may help maintain joint homeostasis, likely through FGFR3-mediated anabolic signaling, whereas its loss reveals or amplifies the catabolic functions of high molecular weight FGF2. The latter appears to drive OA pathogenesis via preferential activation of FGFR1. Therefore, the net effect of FGF2 signaling in cartilage depends critically on the relative abundance and activity of these antagonistic isoforms, providing a clear molecular basis for its dual role in OA [252].

FGF7 is upregulated in OA cartilage and meniscal cells, and intra-articular injection of recombinant FGF7 induces OA-like changes in mice, including cartilage destruction, osteophyte formation, and synovitis. FGF7 promotes cartilage destruction by upregulating matrix-degrading enzymes (MMP3, MMP13, ADAMTS5) and downregulating cartilage extracellular matrix molecules (SOX9, type II collagen, aggrecan) via the FGF7-FGFR1 axis [253].

FGF8 is upregulated in inflamed and hyperplastic synovium following joint injury and contributes to OA pathogenesis by promoting the release of MMP-3 and prostaglandin E2, accelerating extracellular matrix degradation and cartilage breakdown [254]. FGF8 upregulates MMP2 and MMP9 expression and activity in chondrocytes via NF-κB p65-dependent signaling [255]. In animal models of OA, neutralization of FGF8 alleviates ECM loss, highlighting its role as a key mediator of synovitis and cartilage degeneration [254].

FGF9 expression is reduced in OA chondrocytes but is ectopically upregulated at sites of osteophyte formation [256]. Intra-articular delivery of exogenous FGF9 protects cartilage integrity in post-traumatic OA, as shown by reduced collagen X and MMP13 expression together with enhanced collagen II synthesis in both murine destabilization of the medial meniscus (DMM) models and IL-1β–induced ex vivo human cartilage cultures [256]. Notably, FGF9 simultaneously aggravates osteophyte formation, accompanied by increased SOX9 and collagen II expression and enhanced chondrocyte proliferation at osteophyte initiation sites [256].

Sprifermin (recombinant FGF18) demonstrated a favorable safety profile and dose-dependent structural benefits in preserving knee cartilage thickness in OA, despite no significant effect on central compartment cartilage loss [257]. Sprifermin promotes chondrocyte proliferation, restores a hyaline-like phenotype, and improves collagen II:I ratios via intermittent exposure and ERK activation, supporting its anabolic role in OA cartilage regeneration [258]. Furthermore, research indicates that FGF18 alleviates cartilage degradation in OA by promoting chondrocyte proliferation and migration via PI3K-AKT signaling [259]. It suppresses MMP13 expression, enhances collagen II deposition, and protects chondrocytes from IL-1β-induced apoptosis [259]. Additionally, FGF18 restores mitochondrial function, improves mitochondrial fusion and fission, and reduces reactive oxygen species (ROS) production [259].

Collectively, these findings demonstrate that FGFs act as pivotal regulators in skeletal pathology. Genetic alterations contribute to a spectrum of congenital skeletal syndromes, while aberrant FGF signaling in adulthood modulates cartilage degradation, bone remodeling, and osteophyte formation in OA.

### Nervous system diseases

FGFs play central roles in maintaining neuronal integrity, synaptic plasticity, and glial support, thereby influencing brain excitability and cognitive function. Dysregulation of FGF signaling contributes to epilepsy, neurodegenerative disorders, and mood disturbances through altered neuronal activity, impaired inhibitory circuitry, neuroinflammation, and mitochondrial dysfunction. By modulating neurogenesis, cell survival, and neural network stability, FGFs emerge as promising targets for both prevention and treatment of neurological diseases.

Epilepsy is a chronic neurological disorder caused by abnormal neuronal discharge in the brain, with complex pathogenic mechanisms that remain incompletely elucidated. Members of the FGF family have also been found to be closely associated with seizure occurrence and alleviation. The relationship between FGF2 and epilepsy has been extensively reported [260]. FGF2 overexpression increases neuronal excitability and seizure susceptibility but reduces seizure-induced cell death [261]. In an injury-associated epilepsy model, local intrahippocampal FGF2 supplementation promoted neurogenesis, reduced neuronal loss, and decreased the frequency of spontaneous seizures [262]. FGF7 and FGF22 exhibit a synergistic relationship and also play important roles during seizure activity. FGF7 knockout mice are more susceptible to seizures compared to controls, whereas FGF22 knockout mice show resistance to pentylenetetrazol-induced seizures [263]. Other studies have shown that FGF7 knockout mice exhibit epilepsy-associated pathological changes even without chemical induction, including mossy fiber sprouting and enhanced neurogenesis [135]. Moreover, knockout of FGF9 in GABAergic neurons leads to seizures and neuronal apoptosis, suggesting its critical role in inhibitory neuron function and neural network stability. FGF9 deficiency can induce inflammatory responses, neuronal apoptosis, and abnormal glial activation—changes closely associated with seizure activity and neurodegeneration [264]. FGF13 is involved in the pathogenesis of temporal lobe epilepsy by regulating microtubule dynamics and neuronal excitability [265], and interneuronal FGF13 modulates seizure susceptibility through sodium channel-independent mechanisms [266]. In recent years, FGF12 gene mutations have also been implicated in seizure disorders [267, 268].

Alzheimer's disease (AD) and Parkinson's disease are two common age-related neurodegenerative disorders, with several FGF family members reported to be associated with these conditions. FGFs can modulate the cerebral microenvironment, protect neurons, promote neural stem cell proliferation, differentiation, and migration, and facilitate axonal growth and neural tissue maintenance and repair. Multiple FGFs have demonstrated neuroprotective effects capable of ameliorating neurodegeneration in these diseases. FGF2 restored hippocampal function in AD mouse models and holds therapeutic significance for neurocognitive impairment [133]. FGF10 ameliorates neurodegeneration in mouse and cellular models of Alzheimer's disease via reducing tau hyperphosphorylation and neuronal apoptosis [219]. FGF20 mutations have also been associated with Parkinson's disease [269]. Furthermore, FGF21 ameliorates neurodegeneration in rat and cellular models of Alzheimer's disease [270]. FGF21 also possesses important protective effects in Parkinson's disease. Recent studies have found that exercise stimulates the liver to produce substantial amounts of FGF21, which enters the brain through the bloodstream to exert neurovascular protective effects [271]. FGF21 confers neuroprotection in Parkinson's disease by activating the FGFR1-SIRT1 pathway to maintain mitochondrial homeostasis [272]. In diabetic and aged mice, FGF21 has been shown to reduce neuroinflammation and oxidative stress, thereby attenuating neurodegeneration and enhancing protection of neuronal mitochondria through modulation of NF-κB and AMPKα/AKT pathways [273]. Additional research has found that FGF21 protects dopaminergic neurons in Parkinson's disease models via repression of neuroinflammation and modulation of gut microbiota and metabolic homeostasis [219, 274]. Beyond FGF21, neuronal FGF13 inhibits mitochondria-derived damage signals to prevent neuroinflammation and neurodegeneration in a mouse model of Parkinson's disease [275]. Genetic polymorphisms of FGF20 are also considered to be associated with high risk of Parkinson's disease [276], and intracerebroventricular injection of FGF20 improves motor performance in Parkinson's disease mouse models [277].

Major depressive disorder is a common chronic psychiatric condition characterized primarily by alterations in mood, cognition, behavior, sleep, and appetite, potentially leading to impaired overall social functioning. Numerous studies have indicated that certain members of the FGF system are associated with depression. FGF2 is an endogenous anxiety suppressor; postmortem human studies have demonstrated reduced expression of FGF2 in the brains of depressed patients [278]. Both basic and clinical studies have reported decreased FGF2 expression in the prefrontal cortex of depressed patients or rodents exposed to stress conditions, with elevated expression following antidepressant treatment [279]. FGF2 is also required for the antidepressant effects of fluoxetine [280], and administration of FGF2 can alleviate depressive symptoms [281]. A recent intriguing study showed that FGF2 is sensitive to environmental conditions; modifiable lifestyle factors such as physical exercise can increase brain FGF2 expression and improve blood–brain barrier (BBB) function, thereby alleviating anxiety- and depression-like behaviors in male mice [282]. Conversely, FGF9 expression is increased in certain brain regions (e.g., hippocampus, frontal cortex) of depressed patients [283]. Exogenous FGF9 administration exacerbates anxiety- and depression-like behaviors, whereas lentiviral vector-mediated reduction of endogenous FGF9 expression in the dentate gyrus decreases anxiety-like behavior in rats, indicating that unlike FGF2, FGF9 is a novel negative regulator of affect [284]. Additionally, FGF21 levels are elevated in patients with major depressive disorder [285]. In contrast, serum FGF22 levels are significantly reduced in depressed patients and negatively correlated with the inflammatory cytokine IL-1β, FGF22 overexpression in mice ameliorates depression-like behavior [286]. FGF22 deficiency leads to depression-like phenotypes in adult mice [287], and serum FGF22 levels are also significantly decreased in depressed patients with mild cognitive impairment [164].

### Respiratory system diseases

Pulmonary homeostasis depends on precise FGF-mediated regulation of epithelial repair, vascular remodeling, and mesenchymal–epithelial interactions. Aberrant FGF signaling disrupts these processes, contributing to chronic lung diseases, acute injury, fibrosis, and vascular complications. The pleiotropic actions of FGFs across multiple cell types underscore their potential both as biomarkers of disease severity and as therapeutic agents for restoring airway and vascular integrity.

Genetic studies have linked FGFs to multiple pulmonary pathologies. Single nucleotide polymorphisms in *FGF10* are associated with susceptibility to chronic obstructive pulmonary disease (COPD) and with variations in airway branching [288, 289], while SNPs in *FGF3*, *FGF7* and *FGFR4* have been correlated with respiratory distress syndrome (RDS) [290]. Rare *FGF10* mutations have also been identified in lethal pulmonary hypoplasia [291].

In pulmonary arterial hypertension (PAH), FGF2 is consistently overexpressed in remodeled vessels, where it drives smooth muscle cell proliferation and pathological vascular remodeling [292]. Circulating FGF2 levels are elevated in PAH patients and correlate with disease severity, supporting its potential as both biomarker and therapeutic target [293]. Beyond FGF2, FGF1 amplifies endothelin-1 receptor signaling in vascular smooth muscle [294], while reduced FGF10 expression in bronchopulmonary dysplasia exacerbates vascular complications [295]. FGF21 protects endothelial cells against hypoxia-induced apoptosis via PERK–CHOP signaling, highlighting a cytoprotective role in pulmonary vascular disease [296].

In COPD, dysregulated FGF expression is closely tied to airway remodeling. FGF1 is elevated in bronchial epithelium, while FGF2 is upregulated in both epithelial and airway smooth muscle compartments [297, 298]. Exogenous FGF2 administration improves pulmonary blood flow, reduces emphysematous changes, and promotes lung regeneration in elastase-induced models, with clinical trials showing inhaled rFGF2 can alleviate respiratory symptoms [298–300]. Recombinant or gene-delivered FGF7 protects against emphysema by limiting inflammation, protease activity, and epithelial apoptosis [301, 302]. Loss-of-function mutations in *FGF10* further link impaired signaling to COPD susceptibility [303]. Circulating FGF23 levels are elevated in COPD patients and correlate with systemic inflammation and altered mineral metabolism, reflecting its endocrine role in disease progression [304].

FGFs also modulate acute and chronic lung injury. FGF1 attenuates LPS-induced acute lung injury (ALI) by reducing inflammation and oxidative stress [196], and also mitigates TGF-β1–driven pulmonary fibrosis by protecting alveolar epithelial cells, suppressing myofibroblast differentiation, and modulating TGF-β receptor signaling [305]. FGF2 reduces bleomycin-induced mortality and fibrosis and protects against radiation-induced lung injury [306, 307]. Similarly, FGF4 alleviates LPS-induced ALI by suppressing pro-inflammatory mediator production through inhibition of TLR4/NF-κB signaling in macrophages and epithelial cells [308]. FGF10–FGFR2b signaling drives alveolar epithelial regeneration and expansion of mesenchymal progenitors [216]. FGF18 alleviates hyperoxia-induced damage by limiting oxidative stress and inflammation [309], whereas FGF21 protects against LPS-induced injury by suppressing TLR4/MyD88/NF-κB signaling [310].

Collectively, FGFs act at multiple cellular levels to regulate pulmonary homeostasis and disease. Their pleiotropic functions highlight them as promising diagnostic markers and therapeutic targets for respiratory disorders.

### Renal disorders

In the kidney, FGFs govern tissue development, injury repair, and chronic disease progression. Acute kidney injury is characterized by rapid changes in circulating and urinary FGFs that reflect both damage and reparative processes, whereas chronic kidney disease involves sustained dysregulation of endocrine FGFs, particularly FGF21 and FGF23, contributing to mineral metabolism disturbances, bone fragility, and immune dysfunction. These multifaceted roles position FGFs as critical determinants of renal pathology and promising targets for therapeutic intervention.

In acute kidney injury (AKI), circulating and urinary FGF23 levels rise rapidly, particularly after cardiac surgery, and outperform conventional urinary markers in predicting disease onset and prognosis [311, 312]. Clinical studies show that both intact FGF23 (iFGF23) and C-terminal fragments (cFGF23) rise rapidly after cardiac surgery and predict AKI with greater sensitivity than conventional urinary injury markers [313, 314]. High FGF23 levels are also associated with increased mortality risk in critically ill patients, although whether FGF23 directly contributes to poor outcomes remains unclear [315]. Other FGFs show therapeutic potential in renal injury repair. Recombinant FGF10 has been proposed to rescue ureteric branching defects in Fraser syndrome [316]. FGF7 expression is induced after chemically induced renal injury, and administration of recombinant truncated human FGF7 effectively prevents urothelial damage in animal models, suggesting a promising strategy for treating bladder or urinary tract injury [317, 318].

In chronic kidney disease (CKD), endocrine FGFs, particularly FGF21 and FGF23, are critically involved in disease progression. Circulating FGF21 levels rise as early as stage 2 CKD [319]. While FGF21 may exert protective and anti-aging effects, sustained elevation is associated with adverse outcomes, including growth retardation, impaired bone mineralization, enhanced marrow adipogenesis, and the development of CKD–mineral and bone disorder (CKD-MBD) [320, 321].

FGF23 has emerged as one of the earliest detectable biomarkers reflecting disturbances in phosphorus metabolism during the initial stages of CKD [322]. However, chronic elevation suppresses calcitriol production, induces secondary hyperparathyroidism, and promotes CKD-MBD, contributing to bone fragility, ectopic calcification, and increased mortality [323]. Beyond mineral metabolism, FGF23 exerts immunomodulatory effects in CKD [324]. Experimental studies in mice and humans demonstrate that FGF23 disrupts leukocyte recruitment to inflamed tissues by inhibiting chemokine-mediated neutrophil adhesion and migration. Mechanistically, FGF23 binding to FGFR2 activates PKA, suppresses Rap1 signaling, and thereby prevents β2 integrin activation. Neutralization of FGF23 or downregulation of FGFR2 restores leukocyte recruitment and host defense. These findings highlight FGF23 as a direct regulator of innate immunity, contributing to infection susceptibility in CKD [324].

### Cardiovascular disorders

FGFs modulate adaptive and maladaptive cardiac and vascular responses, regulating cardiomyocyte survival, angiogenesis, and hypertrophic remodeling. Following myocardial infarction or pressure overload, specific FGFs provide cytoprotection, limit oxidative stress, and stimulate tissue regeneration, while others fine-tune calcium handling and cardiomyocyte proliferation. These context-dependent effects highlight FGFs as key mediators of cardiovascular homeostasis and potential therapeutic candidates for ischemic and hypertrophic heart disease.

In myocardial infarction (MI), FGFs such as FGF1, FGF2, FGF7, FGF9, and FGF21 are involved in adaptive responses, including the restoration of cardiac contractile rate and reduction of infarct size.

FGF1 exhibits cardioprotective effects against MI by reducing infarct size and improving post-ischemic cardiac function [325]. However, its interaction with heparin, a standard therapy for MI patients, can diminish these protective effects. A modified FGF1 variant with reduced heparin-binding affinity (FGF1DHBS) has been developed, which demonstrates enhanced cardioprotective efficacy even in the presence of heparin. This variant not only preserves the cardioprotective signaling of FGF1 but also shows improved tissue distribution to the heart, making it a promising therapeutic agent for ischemic heart disease [325].

Endogenous FGF2 is vital for both the early and long-term cardiac responses to ischemia–reperfusion injury, reducing infarct size, preserving contractile function, and promoting hypertrophic and angiogenic adaptation in the peri-infarct area [326]. FGF2 protects against myocardial infarction by enhancing HIF-1α accumulation, thereby limiting ischemic damage [327].

FGF7 attenuates post-infarction dysfunction by reducing oxidative stress, inhibiting cardiomyocyte apoptosis, and activating the PI3Kα/AKT–NRF2 axis to suppress ROS production [328]. FGF9 improves survival and ventricular function following infarction by stimulating endothelial cells, increasing microvascular density, promoting compensatory hypertrophy, and reducing fibrosis [329].

FGF21 plays a crucial role in the liver-heart cross-talk after MI. The IL-6/STAT3/MR/FGF21 axis mediates this communication, with FGF21 exerting protective effects against MI. FGF21 expression is negatively regulated by the mineralocorticoid receptor (MR) in hepatocytes. MR deficiency or its blockade by spironolactone increases FGF21 levels, enhancing cardiac protection. The IL-6/STAT3 pathway activated in the liver after MI suppresses MR expression, leading to increased FGF21 production. Clinically, spironolactone increases plasma FGF21 levels and improves cardiac function in patients with heart failure due to MI [330].

Other FGFs contribute through distinct mechanisms. FGF13 regulates calcium handling by modulating microtubule stability; its deficiency restores contractility and calcium transients in pressure-overload heart failure models [331]. FGF16, regulated by GATA4, supports cardiomyocyte proliferation, and its dysregulation is implicated in atrial septal defect, with overexpression rescuing proliferation defects in patient-derived cardiomyocytes [332]. FGF20 exerts antihypertrophic effects under stress by activating the SIRT1–FOXO1 pathway, enhancing antioxidant defense and mitigating oxidative stress [333].

### Metabolic disorders

Endocrine FGFs, including FGF19, FGF21, and FGF23, integrate systemic nutrient sensing, energy homeostasis, and inter-organ communication. Dysregulated FGF signaling underlies metabolic disorders such as obesity, type 2 diabetes, nonalcoholic fatty liver disease, and chronic kidney disease by impairing lipid and glucose metabolism, insulin sensitivity, and phosphate balance. Modulating these pathways offers promising avenues for restoring metabolic equilibrium and preventing disease progression.

FGF19 is a hormone produced in enterocytes that plays a crucial role in regulating hepatic bile acid, lipid, and carbohydrate metabolism. In nonalcoholic fatty liver disease (NAFLD), serum FGF19 levels are significantly reduced, indicating impaired FXR-mediated and FGFR4-mediated signaling [334]. This reduction in FGF19 contributes to the pathogenesis of NAFLD by disrupting the normal regulatory mechanisms of bile acid synthesis and metabolism. Conversely, in lean NAFLD patients, serum FGF19 levels are higher compared to non-lean NAFLD patients, suggesting a potential compensatory mechanism or differential metabolic adaptation. This difference in FGF19 levels between lean and non-lean NAFLD patients may explain the distinct metabolic and histological profiles observed in these subgroups [335]. Targeting FGF19 and its associated signaling pathways offers a promising therapeutic strategy for managing NAFLD.

FGF21 has emerged as an important regulator of metabolism, offering therapeutic potential for obesity, type 2 diabetes, and hepatic steatosis. In humans, serum FGF21 levels elevated in overweight/obese individuals and correlated with adiposity, insulin, and triglycerides, while inversely related to high-density lipoprotein (HDL) cholesterol [336]. In high-fat diet-induced obese (DIO) mice, FGF21 treatment reduces body weight and fat mass by increasing energy expenditure and physical activity. Additionally, FGF21 lowers plasma glucose, insulin, cholesterol, and triglyceride levels, thereby improving metabolic health. A key mechanism involves the inhibition of hepatic lipogenic and glucose production pathways, specifically through the downregulation of SREBP-1 and its target genes involved in fatty acid and triglyceride synthesis. FGF21 also enhances hepatic and peripheral insulin sensitivity independently of weight reduction [337]. These findings suggest that FGF21 can ameliorate multiple metabolic disorders and could serve as a powerful therapeutic agent for metabolic diseases.

FGF23 regulates phosphorus metabolism and is a strong predictor of mortality in dialysis patients. It serves as an early biomarker of disordered phosphorus metabolism in the initial stages of CKD. In patients with CKD stages 2–4, serum FGF23 levels are elevated, even when serum phosphate and PTH levels are normal [322]. Elevated FGF23 is also associated with adverse outcomes in CKD, including left ventricular hypertrophy, heart failure, atrial fibrillation, anemia, and increased mortality. FGF23 excess may contribute to the pathophysiology of CKD complications, making it a potential therapeutic target for improving clinical outcomes in CKD patients [338].

### Cancer

Aberrant activation of the FGF/FGFR axis has been documented across diverse malignancies, where they contribute to tumor progression by promoting cell proliferation and survival, enhancing invasive and migratory capacity, and stimulating angiogenesis [49, 339].

In terms of tumor growth and survival, FGF2 is one of the most extensively studied ligands. FGF2 drives the conversion of normal stem cells into cancer stem cells, conferring stemness, tumorigenicity, and malignant growth. It promotes survival and activates autocrine FGFR–PI3K/AKT signaling, positioning FGF2 as a potent initiator of tumorigenesis in the inflammatory microenvironment [340]. Increased expression of FGF4 has been observed in germ cell tumors and ovarian cancer stem-like cells, where it augments tumor-initiating potential [341]. FGF5 has been shown to facilitate proliferation and metastasis of hepatocellular carcinoma (HCC), and its suppression by miR-188-5p markedly reduces malignant growth [342]. FGF8 is overexpressed in prostate cancer, correlates with advanced stage and poor prognosis, persists in androgen-independent disease, and promotes tumor growth [343]. FGF9 promotes prostate cancer initiation, progression, and bone metastasis by inducing reactive stroma through c-Jun–mediated upregulation of TGFβ1 signaling, thereby enhancing tumor growth and metastatic potential [344]. FGF9 is upregulated in NASH-driven hepatocellular carcinoma, where it promotes extracellular matrix accumulation and tumor formation via ERK1/2–GSK3β–mediated stabilization of β-catenin [345]. FGF19 is particularly notable, as gene amplification and overexpression drive tumorigenesis in prostate cancer, hepatocellular carcinoma, and head and neck squamous cell carcinoma [346–348]. More recently, FGF23 has been implicated in prostate cancer, where it enhances proliferation, invasion, and anchorage-independent growth in vitro, while knockdown reduces tumor burden in vivo [349].

FGFs also play critical roles in invasion and migration. Tumor–stroma interactions can activate fibroblasts to secrete FGFs, thereby fostering a more invasive phenotype. Nuclear translocation of FGF2 has been linked to pancreatic cancer invasiveness, with its inhibition markedly reducing invasive behavior [350]. In ovarian cancer, cancer-associated fibroblast (CAF)-derived FGF7 stabilizes HIF-1α to drive epithelial–mesenchymal transition (EMT), thereby enhancing proliferation, migration, and invasion. High FGF7 correlates with advanced disease and poor prognosis, while its neutralization suppresses tumor growth [351]. In gastric cancer, FGF7 enhances invasion and migration by activating the PI3K/Akt/mTOR pathway to upregulate THBS1 [352]. In oral squamous cell carcinoma (OSCC), FGF8 is upregulated and correlates with poor clinical parameters. Functionally, FGF8 enhances invasion and metastasis by inducing EMT, underscoring its pro-metastatic role in OSCC progression [353]. Cancer-associated fibroblast–derived FGF9 enhances gastric cancer cell survival and invasiveness [354]. In pancreatic cancer, stromal FGF10 promotes tumor cell migration and invasion by upregulating MT1-MMP and TGF-β1, leading to poor prognosis [355]. FGF18 promotes ovarian cancer metastasis by activating NF-κB signaling, enhancing angiogenesis and M2 macrophage infiltration, and is associated with poor prognosis [356].

Angiogenesis represents another major oncogenic function of FGFs, with FGF1 and FGF2 being the most prominent drivers. FGF1 promotes tumor angiogenesis and cancer cell survival in high-grade serous ovarian cancer, where its amplification correlates with increased CD31⁺ vascular density and poorer overall survival [357]. FGF2 plays an essential autocrine role in melanoma cell survival, growth, and angiogenesis, where its overexpression enhances tumorigenicity and promotes aberrant vessel formation [358]. FGF2 synergizes with PDGF-BB to drive tumor angiogenesis and metastasis by inducing reciprocal responsiveness between endothelial and mural cells, resulting in disorganized neovascularization that facilitates tumor progression [359].

## Therapeutic targeting of the FGF axis

### FGF-based therapies

FGF-based therapies mainly include FGF1, FGF2, FGF7, FGF18, FGF19, and FGF21, which play important roles in metabolic regulation, epithelial repair, and bone and cartilage regeneration. These factors have demonstrated promising therapeutic potential in conditions such as insulin resistance, oral mucositis, osteoarthritis, and liver metabolic disorders. Overall, FGF-targeted strategies represent a well-established and rapidly evolving approach in regenerative and metabolic medicine (Table 1).

**Table 1:** Summary of clinical strategies targeting the FGF axis

Category	Specific agent	Key target	Indication	Clinical progress/key considerations	Phase
**FGF-Based Protein Therapies**	FGF1 (Wild-Type)	FGFR	Diabetes and its complications (protective against diabetic nephropathy and cardiomyopathy)	Metabolic regulation, improves insulin sensitivity, glycemic control, and nutrient stress adaptation	Phase1(NCT00425178, Completed)
	FGF2 (Trafermin)	FGFR	Periodontal regeneration, skin ulcers (Approved in Japan)	Tissue repair and regeneration	Phase3 (NCT01015404, Completed)
	FGF7 (Palifermin)	FGFR2b	Prevention and management of chemotherapy-induced oral mucositis (FDA Approved)	Epithelial proliferation and repair	Approved
	FGF18 (Sprifermin)	FGFR3	Knee osteoarthritis	Chondrocyte proliferation and differentiation	Phase2 (NCT01919164, Completed)
	FGF19 engineered variant (e.g., NGM282)	FGFR4β-Klotho	Primary Sclerosing Cholangitis (PSC), NASH	Metabolic regulation	Phase2 (NCT02704364, NCT02443116, NCT03912532 Completed)
	FGF21 engineered variants: LY2405319	FGFRβ-Klotho	Obesity and diabetes	Metabolic regulation	Phase1 (NCT01869959, Completed)
	Long-acting FGF21 analogs (Pegbelfermin, Pegozafermin, Fc-fusions)	FGFRβ-Klotho	Metabolic disorders	Pegbelfermin (BMS-986036): PEGylated, high immunogenicity; Pegozafermin (BIO89-100): Glyco-PEGylation; Fc-fusions (e.g., Efruxifermin): Markedly prolong bioactivity	Pegbelfermin: Phase2 (NCT03486899, NCT03486912, Completed) Pegozafermin: Phase3 (NCT06419374 Recruiting) Efruxifermin: Phase3 (NCT06528314, NCT06215716, Recruiting)
	FGF21 receptor agonists (C3201, BFKB8488A, MK-3655)	Targets FGFR1/β-Klotho complex directly	Metabolic disorders	Albumin-fused antibodies or monoclonal antibodies; demonstrated promising translational potential	BFKB8488A: Phase1 (NCT03060538, Completed)
	FGF23 Targeting (Burosumab)	Binds and neutralizes FGF23 activity	X-linked Hypophosphatemia	Effectively increases serum phosphate, improves rickets	Approved
	FGF23 small molecules/peptides	Blocks FGF23-FGFR/α-Klotho interaction	Hypophosphatemia	Increases serum phosphate, enhances renal phosphate reabsorption, improves skeletal abnormalities and quality of life	Preclinical
**Small-Molecule FGFR Inhibitors**	First-Generation: Multi-Kinase Inhibitors (Derazantinib/ARQ 087, Dovitinib/TKI258, Nintedanib/BIBF1120, Lucitanib/E3810, Lenvatinib, Regorafenib, Brivanib, Ponatinib, Orantinib, Sunitinib, Cediranib)	FGFR & VEGFR, PDGFR, KIT, etc	Various Cancers (Some approved for other indications)	Broader kinase spectrum, complex pharmacodynamics, higher toxicity	Derazantinib: Phase1 (NCT04098692, Completed) Dovitinib: Phase2, 3 (NCT02116803, Completed) Nintedanib: Phase2 (NCT01788982, Completed) Lucitanib: Phase3 (NCT02135107, Completed)
	Second-Generation: Selective FGFR Inhibitors				
	Pan-FGFR Inhibitors: AZD4547	potently and selectively targets FGFR1, FGFR2, and FGFR3 kinase activities	Gastric, lung, bladder, and hormone receptor–positive breast carcinomas (with FGFR amplification/mutation)	First-generation selective inhibitor, Demonstrated clinical responses in Phase 2 trials	Phase2,3 (NCT02965378, Completed)
	Pan-FGFR Inhibitors: Erdafitinib	Oral pan-FGFR tyrosine kinase inhibitor	FGFR-altered urothelial carcinoma, multiple solid tumors	FDA approved; Phase 3 THOR trial confirmed superiority over chemotherapy in metastatic urothelial carcinoma	Approved
	Pan-FGFR Inhibitors: Futibatinib	Oral, covalently binding and irreversible pan FGFR1–4 inhibitor	Intrahepatic Cholangiocarcinoma (iCCA) with FGFR2 fusions or rearrangements	Capable of overcoming resistance associated with ATP competitive FGFR inhibitors	Approved
	FGFR1/2/3/4 Inhibitors: Pemigatinib	Oral small-molecule inhibitor with high selectivity towards FGFR1, FGFR2, and FGFR3	Cholangiocarcinoma (with FGFR2 fusion or rearrangement)	first targeted treatment for this indication (ORR 35.5% in FIGHT 202)	Approved
	FGFR1/2/3/4 Inhibitors: Infigratinib (BGJ398)	Potent, orally bioavailable, selective inhibitor of FGFR1, FGFR2, FGFR3, and FGFR4	Cholangiocarcinoma (with FGFR2 fusion/rearrangement), urothelial carcinoma, achondroplasia (lower doses)	FDA approved (ORR 23.1%), also in development for achondroplasia	Approved
	Isoform-Selective Inhibitors:			Designed to mitigate off-target toxicities of pan-FGFR inhibition	
	FGFR2-Selective: RLY-4008 (Lirafugratinib)	FGFR2 (Highly Selective)	Advanced solid tumors harboring FGFR2 fusions/alterations (e.g., iCCA)	Rational design achieved high selectivity, mitigating off-target toxicities	Phase1,2 (NCT04526106, Active, not recruiting)
	FGFR3-Selective: TYRA-300	FGFR3 (Highly Selective)	FGFR3-altered urothelial carcinoma, achondroplasia	Designed to sustain activity against common FGFR3 resistance mutation (V555M); mitigates FGFR1-mediated off-target toxicities	Phase2 (NCT07265947, NCT06842355, Recruiting)
	FGFR4-Selective: Fisogatinib (BLU-554)	FGFR4 (Highly Selective)	Hepatocellular Carcinoma (HCC) with FGF19 overexpression	Specifically disrupts the FGF19-FGFR4 oncogenic axis	Phase1,2 (NCT04194801, Completed)

Trial status and phase are per ClinicalTrials.gov

FGF1, traditionally recognized for its mitogenic activity, has emerged as a metabolic regulator with therapeutic potential in diabetes and its complications [360]. It improves insulin sensitivity, glycemic control, and nutrient stress adaptation, while also exerting protective effects against diabetic nephropathy, preserving myocardial integrity, and preventing diabetic cardiomyopathy [199, 361, 362]. Despite these benefits, the strong mitogenicity of wild-type FGF1 raises concerns for long-term use, underscoring the need for safer FGF1 variants in clinical applications [360]. Recent studies identify hepatocyte-derived FGF1 as a key protector against drug-induced liver injury. By signaling through FGFR4–ERK1/2 to repress HNF4α-driven bile acid synthesis, FGF1 restores metabolic homeostasis. This highlights the therapeutic potential of engineered, non-mitogenic FGF1 analogs for treating cholestatic and metabolic liver disorders [197].

FGF2 facilitates periodontal regeneration by promoting the coordinated formation of cementum, periodontal ligament, and alveolar bone, as consistently shown in animal models of surgically induced defects [363]. The recombinant FGF2 derivative Trafermin has been developed and clinically applied, receiving approval for the treatment of skin ulcers, thereby underscoring the broader therapeutic potential of FGF2 in tissue repair and regeneration [364].

FGF7 plays key roles in epithelial cell proliferation, tissue repair, and maintenance of mucosal barrier integrity. Based on these biological functions, its recombinant form, palifermin, became the first FDA-approved FGF-derived therapeutic and is currently used to prevent and manage oral mucositis induced by cancer therapies, thereby reducing treatment-related toxicities and improving patients’ quality of life [365]. A recent study identifies FGF7 as a key factor that promotes tendon regeneration and suppresses fibrosis by directing tendon progenitor cell fate and inhibiting a profibrotic lineage, with a sustained-release GelMA hydrogel system demonstrating therapeutic potential for functional tendon repair [211]. Furthermore, ongoing efforts to optimize the stability and production of palifermin are expected to broaden its potential applications in wound healing, tissue engineering, and other regenerative medicine contexts.

Recombinant FGF18 (sprifermin) has emerged as a promising candidate for disease modification in knee OA. In clinical trials, sprifermin has been associated with dose-dependent reductions in total and lateral femorotibial cartilage loss, cartilage volume decline, and joint space narrowing, despite limited effects on central medial compartment cartilage thickness [257]. Long-term data from the FORWARD (FGF-18 Osteoarthritis Randomized Trial with Administration of Repeated Doses) study further support sustained structural benefits, with improvements in cartilage maintained for at least 3.5 years post-treatment, alongside clinically meaningful pain reduction in high-risk subgroups [366]. Across studies, sprifermin demonstrated a favorable safety profile without major adverse events [257, 366]. A recent advance in FGF18-based osteoarthritis therapy demonstrates a shift from recombinant protein delivery to sustained genome-level activation via a CRISPR/Cas9-mediated system. The study developed chondrocyte-targeted hybrid exosomes (CAP/FGF18-hyEXO) to efficiently deliver an FGF18-editing tool in vivo. These exosomes were further encapsulated in lubricating hyaluronic acid microgels (CAP/FGF18-hyEXO@HMs), which provide both sustained gene editing and self-renewable hydration lubrication. This combined strategy synergistically enhances cartilage regeneration, reduces inflammation, and mitigates disease progression in preclinical OA models [367]. Collectively, these findings highlight FGF18 as a promising therapeutic candidate capable of modifying disease progression and improving clinical outcomes in OA.

FGF19 has been extensively engineered to enhance its therapeutic potential while minimizing adverse effects. Structure-guided modification of FGF19 has led to variants that retain favorable metabolic activity but exhibit reduced mitogenic properties, thereby lowering the risk of tumorigenesis. Among these, NGM282 (M70) has emerged as a leading candidate, demonstrating preservation of bile acid regulation without proliferative activity through suppression of the STAT3 pathway [368]. Clinical trials have shown that NGM282 provides beneficial effects in patients with primary sclerosing cholangitis and NASH [369, 370]. Beyond engineered variants, pharmacological strategies to induce endogenous FGF19 expression, including the farnesoid X receptor (FXR) agonists obeticholic acid and Px-104, have progressed into clinical testing and support their potential use in cholestatic liver diseases as well as nonalcoholic fatty liver disease [25]. Collectively, these studies underscore FGF19 as a promising therapeutic avenue for metabolic and hepatobiliary disorders.

Engineered FGF21 variants have been extensively developed to improve stability, pharmacokinetics, and therapeutic efficacy. LY2405319, generated through structural optimization, demonstrated comparable efficacy to native FGF21 in lowering glucose, insulin, and body weight in preclinical studies, and improved metabolic profiles in obese and diabetic patients during phase I trials [371, 372]. To overcome the limitations of native FGF21, such as rapid clearance and poor stability, several engineering strategies have been pursued to generate long-acting analogs. PEGylation represents an early approach, for instance, pegbelfermin (BMS-986036) has advanced to phase 2b clinical trials, although its use has been associated with high immunogenicity and anti-drug antibody formation [373–375]. A next-generation molecule, pegozafermin (BIO89-100), utilizes glyco-PEGylation technology and has demonstrated improved pharmacokinetics and dosing convenience in clinical studies [376–378]. Another strategy is the design of Fc-fusion proteins, which extend circulating half-life by conjugation with antibody scaffolds. Representative Fc-fusion molecules, including PF-05231023, efruxifermin (EFX, also known as AKR-001), and BOS-580, have all demonstrated favorable safety and metabolic efficacy in early clinical trials [379–381]. Overall, Fc-fusion approaches markedly prolong bioactivity but require careful evaluation of immunogenicity and long-term safety. In addition, structural insights into the FGFR1/β-Klotho complex have enabled the rational design of FGF21 receptor agonists [382]. Examples include the albumin-fused antibody C3201 and monoclonal antibodies directly targeting FGFR1c/KLB, among which BFKB8488A and MK-3655 have entered clinical development and demonstrated promising translational potential [383–385].

FGF23 represents a critical therapeutic target in phosphate metabolism and hypophosphatemic disorders. Preclinical studies have shown that FGFR inhibitors such as PD173074 and NVP-BGJ398, as well as the MAPK pathway inhibitor PD0325901, can reduce FGF23 activity, although their broad effects limit clinical application [386, 387]. More selective approaches, including FGF23 C-terminal peptides, small molecules that block the FGF23–FGFR/α-Klotho interaction, and monoclonal antibodies against the N- or C-terminal of FGF23, have demonstrated the ability to restore phosphate homeostasis and improve skeletal abnormalities in hypophosphatemia mice [388–390]. Clinically, the human monoclonal antibody burosumab has shown significant efficacy in X-linked hypophosphatemia (XLH), where subcutaneous administration increased serum phosphate, enhanced renal phosphate reabsorption, elevated circulating 1,25(OH)₂D, and improved quality of life, with a generally favorable safety profile [391, 392]. A recent study has identified a novel pathogenic PHEX variant (c.T1349C; p.L450P) in XLH and established a corresponding CRISPR/Cas9 knock-in mouse model that faithfully recapitulates the human disease phenotype. This research further demonstrates, for the first time, that a single intramuscular injection of a minicircle DNA (MC-DNA) vector expressing a competitive N-terminal fragment of FGF23 (aa 180–251) can effectively ameliorate hypophosphatemia, improve bone mineralization, and correct skeletal abnormalities in the mutant mice without observable toxicity, highlighting a novel, safe, and effective non-viral gene therapy strategy for XLH [393].

### FGFR inhibitors

Small-molecule inhibitors targeting FGFRs have been developed for cancers with FGFR alterations, including urothelial, breast, lung, liver, and cholangiocarcinoma [1, 9]. These agents bind to the ATP-binding pocket of the FGFR tyrosine kinase domain and are classified as multikinase (first-generation) or selective (second-generation) inhibitors [1, 9]. Multikinase FGFR inhibitors, including derazantinib (ARQ 087), dovitinib (CHIR258 or TKI258), nintedanib (BIBF1120), lucitanib (E3810), lenvatinib, regorafenib, brivanib, ponatinib, orantinib, sunitinib, and cediranib, act on multiple receptor tyrosine kinases (RTKs) in addition to FGFRs, thereby affecting both tumor cells and the surrounding microenvironment through the regulation of angiogenesis and immune signaling [1, 9]. However, their broad activity results in complex pharmacodynamics and higher toxicity. Selective FGFR inhibitors possess enhanced target specificity and encompass several categories, including pan FGFR inhibitors such as AZD4547, erdafitinib, futibatinib, and rogaratinib [1, 9]; FGFR1–3 inhibitors such as pemigatinib and infigratinib [1, 9]; and isoform-specific inhibitors targeting individual subtypes, including RLY-4008 [394], LHQ490 [395], and BW710 [396] for FGFR2, TYRA-300 [397] for FGFR3, and fisogatinib (BLU554) [398], FGF401 [399], and BLU9931 [400] for FGFR4. Given that multiple selective FGFR inhibitors have received clinical approval, the following section will primarily address this class of agents (Table 1).

#### Pan-FGFR inhibitors

AZD4547 is a first-generation selective FGFR tyrosine kinase inhibitor [401]. It potently and selectively targets FGFR1, FGFR2, and FGFR3, inhibiting their kinase activities and suppressing downstream signaling in tumor cells with deregulated FGFR expression [401]. Preclinical and clinical studies have demonstrated its ability to suppress tumor growth through effective blockade of aberrant FGFR signaling [401, 402]. AZD4547 has been evaluated in phase II trials for several cancers, including gastric, lung, bladder, and hormone receptor–positive breast carcinomas, showing clinical responses in patients with FGFR amplification or mutation [401, 402].

Erdafitinib, an oral pan-FGFR tyrosine kinase inhibitor, has shown significant antitumor activity across multiple FGFR-altered solid tumors [403]. In the RAGNAR study, it demonstrated a 30% objective response rate in 16 tumor types [404]. In the BLC2001 study, erdafitinib achieved a 40% response rate in FGFR-altered urothelial carcinoma [405]. The phase 3 THOR trial further confirmed its superiority over chemotherapy in FGFR-altered metastatic urothelial carcinoma, with longer median overall and progression-free survival [406]. The final analysis of the phase 2 THOR-2 trial demonstrates that erdafitinib is effective in earlier-stage, FGFR-altered non-muscle-invasive bladder cancer. It significantly improved recurrence-free survival versus intravesical chemotherapy in high-risk papillary disease (median not reached vs. 11.6 months; HR 0.28) and achieved high complete response rates (94% in carcinoma in situ; 89% in intermediate-risk disease). The safety profile was manageable, with hyperphosphatemia (76%) as the most common adverse event [407].

Futibatinib is an orally available, covalently binding and irreversible pan FGFR1–4 inhibitor that suppresses aberrant FGFR phosphorylation and downstream oncogenic signaling in FGFR altered tumors [408]. Clinically, futibatinib has demonstrated meaningful antitumor activity in malignancies driven by FGFR genomic abnormalities, with the strongest evidence in intrahepatic cholangiocarcinoma with FGFR2 fusions or rearrangements, which supported its first regulatory approval for previously treated unresectable or metastatic disease in this setting [408]. In addition, futibatinib is under active investigation in multiple FGFR deregulated solid tumors including breast, urothelial, oesophageal and non-small cell lung cancers, indicating its potential as a next generation FGFR targeted therapy capable of overcoming resistance associated with ATP competitive FGFR inhibitors [408]. In a pooled safety analysis of 469 patients across multiple clinical trials, futibatinib demonstrated a manageable safety profile characterized by class-specific adverse events. The most common treatment-emergent AEs included hyperphosphatemia (83.8%), alopecia (33.3%), and diarrhea (32.2%), with most events being mild to moderate in severity (Grade 1–2). Notably, the study established that proactive monitoring and dose modifications effectively managed these toxicities, ensuring treatment continuity while maintaining the drug’s potent antitumor activity against FGFR-altered malignancies [409].

#### FGFR1/2/3/4 inhibitors

Pemigatinib is an orally administered small-molecule kinase inhibitor that exhibits high selectivity toward FGFR1, FGFR2 and FGFR3 [410]. It secured accelerated approval from the US FDA in April 2020, marking its status as the first targeted treatment for adults with previously treated, unresectable, locally advanced or metastatic cholangiocarcinoma characterized by an FGFR2 fusion or other rearrangement [410]. The efficacy underpinning this approval stems from the Phase II FIGHT 202 study, which reported an objective response rate of 35.5% in patients with *FGFR2* fusions or rearrangements [411]. The phase II FIGHT-207 trial evaluated pemigatinib in advanced, FGFR-altered solid tumors. Activity was highest in tumors with *FGFR* fusions or rearrangements (ORR 26.5%). Correlative analyses identified *TP53* co-alterations associated with primary resistance and *BAP1* alterations with improved response. Acquired resistance frequently involved secondary *FGFR* gatekeeper and molecular brake mutations [412].

Infigratinib (BGJ398) is a potent, orally bioavailable, FGFR-specific tyrosine kinase inhibitor that selectively targets and inhibits FGFR1, FGFR2, FGFR3, and FGFR4, thereby suppressing the proliferation of malignant cells driven by FGFR alterations [413]. Its principal therapeutic function is the treatment of previously treated, unresectable locally advanced or metastatic cholangiocarcinoma characterized by an FGFR2 fusion or rearrangement, an indication for which it secured accelerated FDA approval in May 2021 [413]. This approval was substantiated by Phase II trial data showing an objective response rate of 23.1% in this patient cohort [413]. Beyond cholangiocarcinoma, Infigratinib is currently in late-stage clinical development for other FGFR-driven conditions, notably urothelial carcinoma and, utilizing lower doses, achondroplasia [413]. Furthermore, the phase III PROOF302 trial demonstrated the efficacy of adjuvant infigratinib in patients with high-risk, resected urothelial carcinoma harboring *FGFR3* alterations. This randomized, placebo-controlled study met its primary endpoint, significantly improving centrally reviewed disease-free survival. These results provide the first evidence that FGFR3 inhibition in the adjuvant setting can offer clinical benefit for this molecularly defined subgroup, thereby supporting the potential expansion of infigratinib's therapeutic application into earlier-stage urothelial cancer [414].

RLY-4008 (lirafugratinib) is a highly selective and irreversible covalent small molecule inhibitor that primarily functions to specifically target and inhibit the kinase activity of FGFR2 [394]. It was rationally designed to achieve high selectivity, approximately 250-fold over FGFR1 and over 5,000-fold over FGFR4, by exploiting dynamic differences in the P-loops of FGFR subtypes, thereby mitigating the dose-limiting off-target toxicities common to pan-FGFR inhibitors, particularly FGFR1-mediated hyperphosphatemia and FGFR4-mediated diarrhea [394]. RLY-4008's clinical significance lies in its capacity to serve as a precision oncology agent for advanced solid tumors harboring FGFR2 fusions or other alterations, such as intrahepatic cholangiocarcinoma [394].

TYRA-300's principal function is as an oral, highly selective FGFR3 inhibitor designed to mitigate the off-target toxicities associated with inhibiting FGFR1 [397]. By leveraging structural differences between FGFR subtypes, it achieves specific inhibition of FGFR3 [397]. A critical advantage of TYRA-300 is its sustained activity against the common FGFR3 gatekeeper resistance mutation (e.g., V555M), positioning it as a potent therapeutic agent for FGFR3-altered urothelial carcinoma and for the treatment of achondroplasia [397].

Fisogatinib (BLU-554) is a highly potent and selective oral inhibitor of FGFR4 [398]. Its mechanism of action centers on selectively disrupting the FGF19-FGFR4 signaling axis, which is aberrantly amplified and serves as a crucial oncogenic driver in a subset of advanced HCC [398]. Fisogatinib's primary clinical role is the treatment of HCC patients whose tumors exhibit FGF19 overexpression, with clinical trials confirming its efficacy in this specific molecular subgroup [398].

## Challenges and opportunities

This review comprehensively delineates the FGF signaling axis, from its fundamental biology to its clinical translation. A central theme that emerges is the therapeutic duality of the pathway: its physiological roles in development and homeostasis can be harnessed through agonism, while its pathological drivers in cancer and other diseases must be suppressed through antagonism. This paradigm is exemplified by two major therapeutic strategies: FGF-based biologics (e.g., Sprifermin/FGF18, Palifermin/FGF7, engineered FGF1/FGF19/FGF21) that promote regeneration and metabolic health, and selective FGFR inhibitors (e.g., Erdafitinib, Pemigatinib, Futibatinib) that block oncogenic signaling. The clinical challenge, therefore, is not merely to target the FGF axis, but to precisely balance agonism versus antagonism based on disease context.

The FGF family, with its canonical, endocrine, and intracellular subclasses, and their cognate receptors, constitute a sophisticated regulatory network integral to embryogenesis, tissue repair, and metabolic homeostasis. Spatiotemporally controlled expression of canonical/paracrine FGFs (e.g., FGF2, FGF8, FGF9, FGF10) is paramount for organogenesis, including limb patterning, lung branching morphogenesis, nephron formation, and cardiac development. Conversely, the endocrine FGFs (FGF15/19, FGF21, FGF23) function as systemic hormones, coordinating inter-organ crosstalk to regulate bile acid synthesis, glucose/lipid metabolism, and phosphate/vitamin D homeostasis. However, dysregulation of this potent signaling system is a cornerstone of numerous pathologies. Aberrant FGF/FGFR activity underpins congenital syndromes and common acquired disorders such as OA, chronic kidney disease, and cancer. This profound understanding has catalyzed the development of two principal therapeutic strategies: (1) FGF-based protein therapies that harness the regenerative or metabolic properties of ligands (e.g., Sprifermin/FGF18 for OA, Palifermin/FGF7 for mucositis, and engineered variants of FGF1, FGF19, and FGF21 for metabolic diseases); and (2) small-molecule FGFR inhibitors (e.g., Erdafitinib, Pemigatinib, Futibatinib) that effectively block oncogenic signaling in tumors harboring *FGFR* alterations. These advancements firmly establish the FGF pathway as a critical target in the era of precision medicine. Notwithstanding the promising progress, the clinical targeting of the FGF axis faces significant hurdles. First, the context-dependent duality of FGF signaling presents a major challenge. The same FGF ligand can exert opposing effects in different tissues or disease stages. For instance, FGF2 exhibits both chondroprotective and catabolic roles in OA, and its low- and high-molecular-weight isoforms have antagonistic effects on bone mineralization. This pleiotropy complicates the prediction of therapeutic outcomes. Second, achieving target selectivity and managing on-/off-target toxicities remain formidable. The high structural homology among FGFR isoforms makes developing highly selective inhibitors difficult; first-generation pan-FGFR inhibitors frequently cause dose-limiting hyperphosphatemia (via FGFR1 inhibition) and severe diarrhea (via FGFR4 inhibition). Furthermore, the inherent mitogenic potential of ligands like wild-type FGF1 and FGF19 necessitates sophisticated protein engineering to decouple their therapeutic metabolic functions from tumorigenic risks. Third, acquired resistance to FGFR inhibitors is an emerging clinical reality. Tumor cells evade suppression through gatekeeper mutations (e.g., FGFR3 V555M), activation of alternative signaling pathways, or phenotypic plasticity, ultimately limiting the durability of response. Finally, the pathophysiological role of chronically elevated endocrine FGFs, such as FGF23 in CKD, poses the challenge of how to antagonize their detrimental systemic effects without disrupting essential physiological circuits.

Future research should focus on several key avenues to overcome these challenges and fully realize the therapeutic potential of the FGF pathway. First, the next generation of therapeutics must prioritize precision. This entails the development of isoform-specific FGFR inhibitors and the continued rational design of engineered FGF variants with optimized safety profiles, such as non-mitogenic FGF19 analogs (e.g., NGM282) and reduced-heparin-affinity FGF1 mutants (FGF1ΔHBS). Second, a deeper, systems-level understanding of FGF signaling in pathophysiology is crucial. Employing single-cell omics, spatial transcriptomics, and advanced organoid models will unravel the intricate cell-specific signaling networks and crosstalk within the tumor microenvironment and metabolic tissues. This knowledge will inform rational combination therapies, such as co-administering FGFR inhibitors with immune checkpoint blockers or other pathway-specific agents, to circumvent resistance. Third, biomarker-driven patient stratification will be essential for maximizing therapeutic efficacy. Clinical trials should increasingly select patients based on specific *FGFR* genetic alterations, ligand overexpression (e.g., FGF19 in HCC), or circulating endocrine FGF levels to identify those most likely to benefit. Finally, exploring non-canonical FGF signaling and the roles of FGFs in emerging areas like aging, neurodegeneration, and the bone-fat-immune axis will undoubtedly uncover novel biology and untapped therapeutic opportunities.

## Data Availability

Not applicable.
